# Lipophilic Peptide Dendrimers for Delivery of Splice-Switching Oligonucleotides

**DOI:** 10.3390/pharmaceutics13010116

**Published:** 2021-01-18

**Authors:** Haneen Daralnakhla, Osama Saher, Susanna Zamolo, Safa Bazaz, Jeremy P. Bost, Marc Heitz, Karin E. Lundin, Samir EL Andaloussi, Tamis Darbre, Jean-Louis Reymond, Rula Zain, C. I. Edvard Smith

**Affiliations:** 1 Clinical Research Center, Department of Laboratory Medicine, Karolinska Institutet, Karolinska University Hospital Huddinge, 141 86 Huddinge, Sweden; h.nakhleh94@gmail.com (H.D.); safa.bazaz.hidush@ki.se (S.B.); jeremy.bost@ki.se (J.P.B.); karin.lundin@ki.se (K.E.L.); samir.el-andaloussi@ki.se (S.E.A.); rula.zain@ki.se (R.Z.); 2 Department of Pharmaceutics and Industrial Pharmacy, Faculty of Pharmacy, Cairo University, Cairo 11562, Egypt; 3 Department of Chemistry and Biochemistry, University of Bern, Freiestrasse 3, 3012 Bern, Switzerland; susanna.zamolo@dcb.unibe.ch (S.Z.); marc.heitz@dcb.unibe.ch (M.H.); tamis.darbre@dcb.unibe.ch (T.D.); jean-louis.reymond@dcb.unibe.ch (J.-L.R.); 4 Department of Biology, Faculty of Science, Cihan University-Erbil, Erbil 44001, Iraq; 5 Centre for Rare Diseases, Department of Clinical Genetics, Karolinska University Hospital, 171 76 Stockholm, Sweden

**Keywords:** splice-switching oligonucleotide, gene therapy, lipophilic, peptide dendrimers, excipients, synergism, transfection, delivery vectors

## Abstract

Non-viral transfection reagents are continuously being developed in attempt to replace viral vectors. Among those non-viral vectors, dendrimers have gained increasing interest due to their unique molecular structure and multivalency. However, more improvements are still needed to achieve higher efficacy and lower toxicity. In this study, we have examined 18 peptide dendrimers conjugated to lipophilic moieties, such as fatty acids or hydrophobic amino acids, that were previously explored for siRNA. Reporter cells were employed to investigate the transfection of single strand splice-switching oligonucleotides (ONs) using these peptide dendrimers. Luciferase level changes reflecting efficiency varied with amino acid composition, stereochemistry, and complexation media used. 3rd generation peptide dendrimers with D-amino acid configuration were superior to L-form. Lead formulations with 3rd generation, D-amino acid peptide dendrimers increased the correction level of the delivered ON up to 93-fold over untreated HeLa Luc/705 cells with minimal toxicity. To stabilize the formed complexes, Polyvinyl alcohol 18 (PVA18) polymer was added. Although PVA18 addition increased activity, toxicity when using our best candidates G 2,3KL-(Leu)_4_ (D) and G 2,3KL-diPalmitamide (D) was observed. Our findings demonstrate the potential of lipid-conjugated, D-amino acid-containing peptide dendrimers to be utilized as an effective and safe delivery vector for splice-switching ONs.

## 1. Introduction

Non-viral vectors are considered attractive tools for delivery of gene therapies. They are advantageous due to their biocompatibility, low immunogenicity, and potential for large-scale production [1,2]. Notwithstanding their encouraging safety, many non-viral transfection reagents are still facing different obstacles, both synthetically and biologically [3].

The first dendritic structures (PPI dendrimer) were reported at the end of the 1970s by Vögtle et al. [4]. This was followed by further development into larger, systemically branched polymeric structures by Tomalia et al. in 1984 [5,6]. Since then, the application of dendrimers has expanded greatly and these compounds have lately gained considerable interest as non-viral transfecting vectors [7,8].

Dendrimers are nano-sized (1–100 nm), highly branched and form three-dimensional structures that consist of three distinct domains: a central core molecule that connects numbers of radiating tree-like branches, an inner shell with multiple branches (dendrons), and an outer shell with terminal functional groups. Each layer of the connected branching units accounts for one complete generation (G) in the dendritic structure and is marked with a generation number. In general, there is a direct relationship between the generation number and dendrimer size.

As transfection reagents, the unique molecular architecture of dendrimers provides them with multiple distinctive properties. Dendrimers have well-defined molecular structures and sizes (10–20 nm) [9] with relatively low polydispersity index (monodispersed with values 0.1–0.3) [10,11] in contrast to linear polymers [12,13]. Additionally, the multivalency offered by the high density of surface groups can be modulated to fit different purposes [14]. Finally, dendrimers are characterized by high buffering capacity that facilitates their escape from endosomes (so-called proton sponge effect) [15]. These properties and others give the dendrimers an advantage compared to other commonly used nanoparticulate delivery systems [16].

A cationic dendrimer such as amine-terminated PAMAM is one of the most used dendritic carriers in biological applications and the first to be used for gene delivery, as well as PPI and PEO dendrimers [17]. Despite their advantages, a major drawback is their toxicity, associated mainly with the chemistry of the surface amine groups. Several efforts were exerted to overcome this issue either by modifying the periphery with neutral groups or by conjugation with negatively charged moieties [18,19,20].

Peptide dendrimers, as the name implies, use amino acids as building blocks in the core, the branches, the dendrimer surface, or any combination of the three units. Peptide dendrimers have been used as protein mimetics, anticancer-, antiallergenic-, antiviral-, and antimicrobial agents as well as in gene delivery [14,21,22,23]. Gene delivery would benefit the most from these vectors, as they provide the necessary positively charged groups to complex with the genetic material, the likelihood to pass the cellular membrane, and the buffering capacity needed to escape endosomes. Despite the mentioned advantages of peptide dendrimers, they are still suboptimal in terms of cellular uptake and endosomal escape, hence affecting their efficiency [24].

Combining different vectors might be a useful strategy to face the multiple biological barriers mentioned previously. This strategy aims to create more stable and functional carriers, while minimizing drawbacks. Lipids are considered as one of the most efficient moieties that can be used with peptide dendrimers. These peptide dendrimer/lipid hybrids proved to achieve synergistic effects in transfection while evading potential toxicities [3,25]. Whereas multivalent interactions between the complexes and the negatively charged cell surface is facilitated by the peptide dendrimer architecture, the presence of lipid is supposed to increase membrane transfer and endosomal escape via fusion properties [3,26,27]. Lipid combinations with polymers have proven to enhance transfection efficiency of plasmid DNA upon complexing PEI dendrimers with different cationic lipids [28,29]. Another successful approach was to combine the lipofectin lipid (combination of cationic (DOTMA) and neutral (DOPE) lipids), with peptide dendrimers to deliver DNA, siRNA, and single stranded ON. It was crucial for activity to have both hydrophobic moieties and charged amino acid groups in complexes of ONs and low generation peptide dendrimers [25,30,31,32,33].

**Figure 1:**
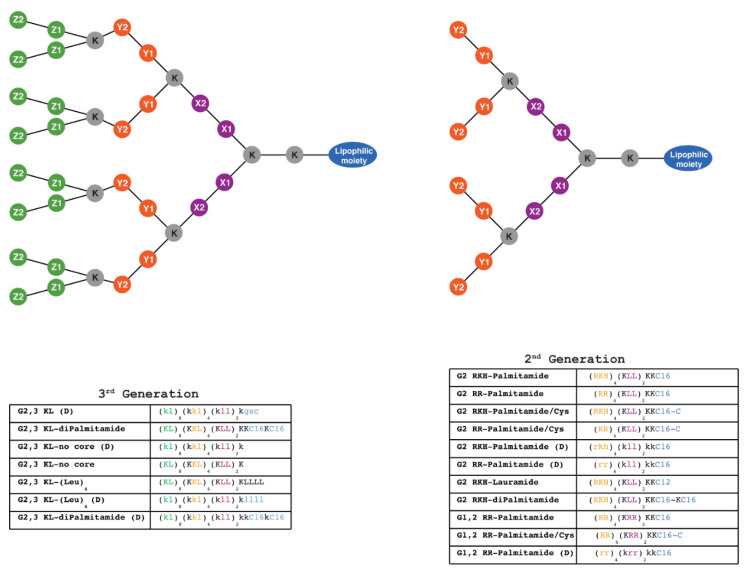
Schematic representation of 2nd and 3rd generation lipophilic peptide dendrimers synthesized using solid-phase chemistry. One-letter code is used to label amino acids forming the dendritic structure. The lipophilic moiety is composed of C12 (Dodecanoic acid), one or two C16 (Hexadecanoic acid), with or without C (Cysteine), or composed of four L (Leucine). K (Lysine) is the branching point for building higher generations, as well as constituting part of the structure and the cationic charge along with, R (Arginine), H (Histidine), and L (Leucine) which are the building units that constitute the rest of the structure. X1, X2, Y1, Y2, Z1, Z2 represent the position of the amino acid with the matching color code (X1 and X2: amino acids in the first layer; Y1 and Y2: amino acids in the second layer; Z1 and Z2: amino acids in the third layer). Capital and small letters indicate L and D stereochemistry of the amino acids, respectively. All dendrimers contain 2 amino acids in the outer layer, except (G2 RKH-Palmitamide, G2 RKH-Palmitamide/Cys, G2 RKH-Palmitamide (D), G2 RKH-Lauramide, and G2 RKH-diPalmitamide) that contain three amino acids. Dendrimers: G 2,3 KL-diPalmitamide, G 2,3 KL-diPalmitamide (D), G 2,3 KL-(Leu)_4_, and G 2,3 KL-(Leu)_4_ (D) were denoted as MH13, DMH13, MH18 and DMH18, respectively, in a previous publication [34].

For peptide dendrimer complexes to have better defined molecular structures, merging the lipid component and the dendrimer into a single entity was desired. This approach was recently demonstrated to provide highly efficient transfection reagents for siRNA and large plasmid DNA [34,35]. Studies with fluorophore labeled dendrimers and oligonucleotides showed that endosome acidification induces protonation of the low p*K*_a_ amino termini of the lipidated dendrimers, which releases part of the dendrimer from the nanoparticle. The latter can then disrupt the endosomal membrane and enable endosome escape [36]. In this study, we synthesized 18 different peptide dendrimers chemically conjugated tolipophilic moieties (fatty acids or hydrophobic amino acids) (Figure 1). We evaluated their ability to transfect single strand splice-switching ONs in serum-containing media. Moreover, the effect of different formulating buffers, the influence of the best performing polymeric excipient from previous studies (PVA 18), the effect of size of peptide dendrimers, their cellular viability, and uptake mechanism were investigated.

## 2. Materials and Methods

### 2.1. Materials

The 18-mer splice-switching ON (5′-CCUCUUACCUCAGUUACA-3′) was synthesized at GE Healthcare (Stockholm, Sweden). The ON has a phosphorothioate backbone and 2′-*O*-methyl modified ribose. Lipofectamine 2000 was purchased from Invitrogen (Stockholm, Sweden).

### 2.2. Cell and Culture Conditions

Luciferase reporter cells representing different tissue origins (HeLa Luc/705, U-2 OS/705, HuH7/705, and Neuro 2a/705 cell) representing (cervical cancer, osteosarcoma, hepatocyte-carcinoma, neuroblastoma cells, respectively) [37], were cultured and maintained in high glucose Dulbecco’s modified Eagle’s medium (DMEM) (Invitrogen, Stockholm, Sweden) supplemented with GlutaMax and 10% FBS (Invitrogen) at 37 °C in a humidified incubator with 5% CO_2_. HeLa Luc/705 was a kind gift from Professor Ryszard Kole, School of Pharmacy of the University of North Carolina, Chapel Hill, NC, USA. The rest of the cell lines were developed in house by Rocha et al [37].

### 2.3. Synthesis and Characterization of Peptide Dendrimers

Solid-phase peptide synthesis (SPPS) was used for the synthesis of the lipophilic peptide dendrimers through Fmoc protection strategy followed by purification using HPLC (Supplementary Materials, Part 2: Synthesis and Characterization: Table S3, and Supplementary Figures S6–18). The protection group was removed before every coupling, as reported before [32,34]. Among the synthesized lipophilic peptide dendrimers, eleven are 2nd generation, and seven are 3rd generation (Figure 1). The dendrimers selection was based on previously reported optimal dendrimers for delivery of splice-switching ON and siRNA [32,34].

### 2.4. ON Transfection of Reporter Cells in Serum-Containing Media

The four reporter cells were seeded at a density of 12 × 10^3^ cells/well, in sterile, clear bottom, white TC (tissue culture) -treated 96-well plates (Sigma-Aldrich, Stockholm, Sweden). The lipid-conjugated peptide dendrimers/ON complexes were formulated by mixing the required amount of the dendrimers (N/P ratio = 20) with the desired amount of ON (final concentration 100 nM) in OptiMEM^®^, HBG (HEPES 20 mM, 5% glucose, pH 7.4), DMEM^®^, or PBS buffers. The formulated complexes were incubated at room temperature for 20 min before adding them to the cells. For complexes containing PVA 18, equal volume of the polymer was directly added after addition of the ON to the dendrimers. The final volume of complex added did not exceed 10% of the cell growth media in the well. L2000 mixture and ON alone were used as a positive and a negative control, respectively. Cells were incubated for 24 h, at 37 °C in a humidified incubator with 5% CO_2_, followed by lysis and luciferase assay analysis.

### 2.5. Luciferase Assay

Luciferase assay protocol reported by Saher et al. [32] was followed with some modifications. Cells were washed with 1× PBS and then lysed with 25 µL of 0.1% Triton-X100 reagent. 5 µL of cell lysates were used to determine protein quantity using the DC Protein Assay (Bio-Rad, Hercules, CA, USA) protocol. The remaining 20 µL of the lysates were mixed with luciferase reagent (Promega, Stockholm, Sweden) automatically by the injector. The RLU of luciferase were determined (GloMax^®^ 96 Microplate Luminometer machine-Promega, Stockholm, Sweden). The final results were represented as fold increase in luciferase activity, calculated by normalizing the RLU values to the total protein quantities and then further normalization against values of untreated cells.

### 2.6. Live Cell Imaging by Fluorescence Microscopy

Selected peptide dendrimers were formulated in OptiMEM^®^, HBG, PBS, and DMEM^®^ and complexed with an Alexa-568-labeled ON with the same chemistry and length as of the above used splice-switching ONs and transfected into HeLa Luc/705 reporter cells were incubated for 24 h, at 37 °C in a humidified incubator with 5% CO_2_, before exchanging the media and rinsing the cells with DMEM^®^ media without Phenol Red (Invitrogen). Live-cell imaging was performed using a fluorescence microscope (Olympus IX81, Olympus America Inc. Center Valley, PA, USA).

### 2.7. Live Cell Imaging by Confocal Microscopy

Complexes of selected peptide dendrimers with an Alexa-568-labeled ON were transfected into HeLa Luc/705 cells. Cells were incubated for 24 h, at 37 °C in a humidified incubator with 5% CO_2_ before adding Hoechst dye (Thermofisher, Stockholm, Sweden), following the manufacturer protocol. Live cell imaging was performed using a confocal microscope (A1R confocal, Nikon, Tokyo, Japan) and analyzed with NIS-Elements software (Nikon, Tokyo, Japan).

### 2.8. Particle Size Measurement

Particle size was measured using Nanoparticle Tracking Analysis (NTA). Each peptide dendrimer was complexed with the ON using the appropriate formulation buffer based on transfection results (OptiMEM^®^, HBG or DMEM^®^). The complexes were diluted with 0.22 µM filtered, freshly prepared PBS, and then injected and measured using the NS500 instrument (NanoSight, Malvern, United Kingdom). Videos (30 s) for the motion of particles were recorded five times per sample. Screen gain and minimum detection threshold were set to 10 and 7, respectively, for processing of the videos.

### 2.9. Cell Viability

Cell viability upon treatment with the lipophilic peptide dendrimers, with or without the polymeric additive (PVA18), was assessed using the cell proliferation assay (WST-1). Cells were seeded and transfected as previously described. The medium was replaced 24 h after treatment with fresh medium supplemented with WST-1 reagent in 1:10 dilution. Cells were then incubated for 2 h in a humidified incubator with 5% CO_2_ at 37 °C. The analysis was performed using (SpectraMAX i3x, Molecular Devices, San Jose, CA, USA). Absorbance was measured at wavelength λ_ex_ = 450 nm, subtracting the background values at λ_em_ = 650 nm. Absorbance values were normalized to the average absorbance of untreated cells.

### 2.10. Data Analysis

All results are shown as mean with the standard error of the mean (SEM). The statistical analysis (GraphPad Prism 6 Software; GraphPad Software, Inc., San Diego, CA, USA) was performed using ANOVA. The post-hoc least significant difference test (Fisher’s LSD) was applied for individual comparisons. *p* < 0.05 was considered significant.

## 3. Results and Discussion

### 3.1. Lipophilic Peptide Dendrimers Surpass Lipofectamine 2000 (L2000) in Transfecting Reporter Cells in Serum-Containing Media

**Figure 2:**
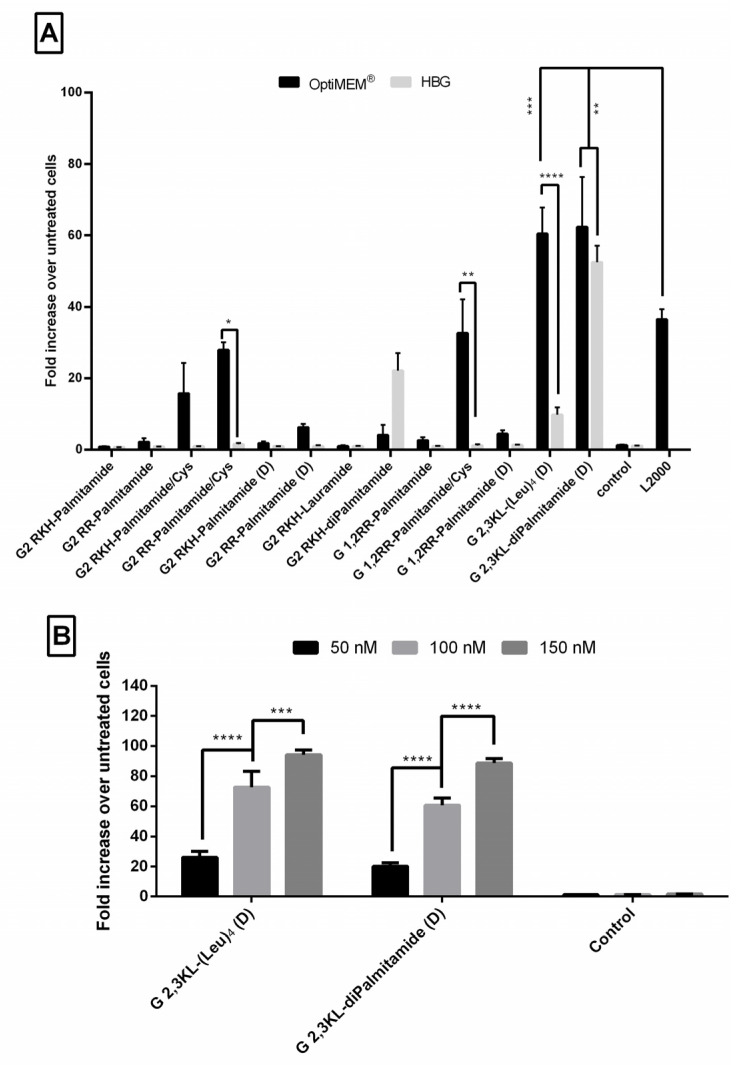
Fold increase in luciferase signals in treated cells over untreated cells after the transfection of the HeLa Luc/705 cells in serum-containing media with lipophilic Peptide Dendrimer/ON complexes. Graphs represent the fold increase in RLUs normalized to the total amount of protein and related to values for untreated cells, for thirteen different dendrimers complexed with ON (N/P ratio = 20). ON complexed with L2000 was used as positive control, while ON in the medium alone acted as a negative control. ON concentration: 100 nM. Luciferase activity was analyzed 24 h after transfection. (**A**) statistical difference between the same complex formulated in OptiMEM^®^ or HBG and compared to L2000. (**B**) A dose response correlation using 3 different doses of the ON (50, 100, 150 nM) complexed with the best performing 3rd generation dendrimers. Each bar represents the mean with the SEM of at least three independent experiments performed in triplicate (*n* ≥ 3). *p*-values were calculated by two-way ANOVA test and the statistical differences was measured using post hoc Fisher’s LSD test. (ns: non-significant, * *p* ≤ 0.05, ** *p* ≤ 0.01, *** *p* ≤ 0.001, and **** *p* ≤ 0.0001).

Luciferase-based assays are frequently used, and considered one of valuable tools to test efficacy of splice-switching ON delivery [38,39]. We have previously utilized such an assay to establish a robust and reliable approach to test the efficacy of a large number of transfection reagents to deliver splice-switching ON [32,40]. The procedure of complex formation was performed according to Saher et al. with some modifications [32]. Unless otherwise mentioned, we used N/P ratio of 20 and a final ON concentration (100 nM). Figure 2A represents 13 different lipophilic peptide dendrimers screened for transfection efficiency. Dendrimer nomenclatures are explained in Figure 1.

As depicted in Figure 2A, ON formulations with 2nd generation lipid dendrimers containing palmitoyl group (C 16) attached to the core showed no activity when L form amino acids were used (G2 RKH-Palmitamide, G2 RR-Palmitamide, and G 1,2RR-Palmitamide). Nevertheless, minimal activity was found for the D form (G2 RKH-Palmitamide (D), G2 RR-Palmitamide (D) and G 1,2RR-Palmitamide (D)). Replacing the palmitoyl group with a shorter lipid did not enhance the activity of RKH based dendrimers (G2 RKH-Lauramide). However, adding an extra palmitoyl group to the core increased the activity for G2 RKH-Palmitamide (D), but only when formulated in HBG.

Interestingly, adding a cysteine residue to the core greatly increased the activity of these dendrimers (G2 RKH-Palmitamide/Cys, G2 RR-Palmitamide/Cys and G 1,2RR-Palmitamide/Cys). These formulations increased luciferase activity, in OptiMEM^®^, but not in HBG buffer, up to 30-fold over the untreated cells. However, the aforementioned transfection efficiency was not significantly different from that of the golden standard L2000. We assume that the increased activity might be due to the formation of dendrimer dimers by disulfide bonding. We also presume that thiol group dimerization would be more feasible in 2nd generation dendrimers due to the lower steric hindrance compared to the 3rd generation versions. However, more experimentations are needed to confirm such a hypothesis.

For the 3rd generation peptide dendrimer (Figure 2A), G 2,3 KL-(Leu)_4_ (D), which has an amino acid hydrophobic core, increased luciferase activity up to 61-fold, when formulated in OptiMEM^®^, but not in HBG (only 10-fold over untreated). It is known that the conformational state of the dendrimers can be affected by the formulating buffer and buffer salt concentration [41,42], thus, affecting complexation, stability, and transfection ability. However, for the G 2,3 KL-diPalmitamide (D) having two C16 lipid tails attached to the core, the level of increase of luciferase activity was comparable in OptiMEM^®^ and HBG buffers (57- and 52-fold, respectively). Both dendrimer formulations (G 2,3 KL-(Leu)_4_ (D) in OptiMEM^®^, and G 2,3 KL-diPalmitamide (D) in OptiMEM^®^ and HBG) displayed significantly higher transfection efficiency in comparison to L2000. (All the results were summarized in a table format (Tables S1 and S2) and added to Supplementary Materials).

To check whether the effect is related to amino acid chirality, two control dendrimers with L amino acid (G 2,3 KL(Leu)_4_ and G 2,3 KL-diPalmitamide) were synthesized (Figure 1). The activity of G 2,3 KL-(Leu)_4_ in L configuration almost disappeared, meanwhile for G 2,3 KL-diPalmitamide (D) the activity was only slightly reduced in L configuration (Supplementary Figure S1). Our data follow the same trend and findings as previously published studies in which the amino acid chirality did affect the siRNA and DNA delivery [34,35,36].

Additionally, other 3rd generation controls were tested and highlighted the importance of having the lipid component in the dendrimer core (Supplementary Figure S1). Previous work confirms that lipids play an integral role regarding the transfection efficiency of peptide dendrimers, possibly because of their ability to facilitate cellular uptake as well as endosomal escape via membrane fusion [30,32,36].

It is understandable that since 2nd generation dendrimers have a lower total positive charge compared to the 3rd generation dendrimers, they are expected to display lower ON complexation, protection, as well as transfecting efficiency [43]. Furthermore, as reported by Haensler and Szoka [44], the higher the generation number of dendrimers, the better their cellular uptake. This can explain the superior transfection efficiency of G 2,3 KL-(Leu)_4_ (D) and G 2,3 KL-diPalmitamide (D) compared to the 2nd generation dendrimers. However, our results are contradictory to the previous findings by Saher et al. [32]. The authors of that study reported that 2nd generation peptide dendrimers (G2RR) were better than the 3rd generation after adding the helper lipid lipofectin. Factors such as lipid type and formulation versus lipid conjugation to the peptide dendrimer underlie such differences.

**Figure 3:**
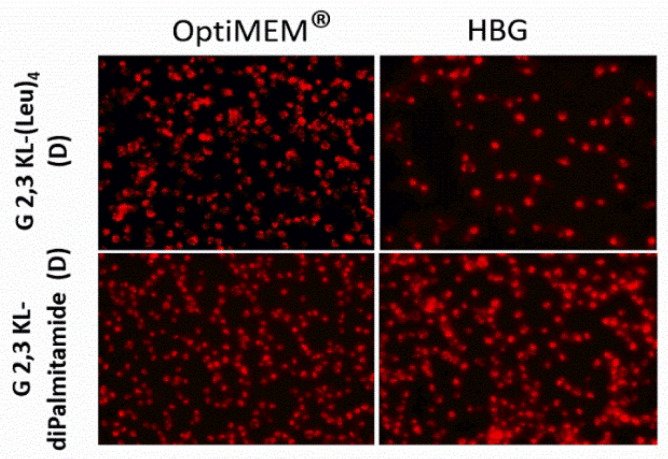
Cellular uptake after transfection in serum-containing media of the selected 3rd generation peptide dendrimers (G 2,3 KL-(Leu)_4_ (D) and G 2,3 KL-diPalmitamide (D)). Dendrimers were formulated in OptiMEM^®^ and HBG, respectively, and complexed with Alexa-568-labelled ON before HeLa Luc/705 transfection in serum-containing media. Live cells were rinsed with DMEM^®^ with no phenol red (Invitrogen), before imaging with the fluorescence microscope (Olympus IX81). (magnification 20×, Scale bar = 100 µm).

A dose-response relationship was established for the best performing 3rd generation peptide dendrimers G 2,3 KL-(Leu)_4_ (D) (in OptiMEM^®^) and G 2,3 KL-diPalmitamide (D) (in HBG), and confirmed that the resulted effect is correlated to the tested ON (Figure 2B). Cellular uptake of the peptide dendrimers/Alexa-568-labeled-ON complexes was then investigated using fluorescence microscopy 24 h after treatment. The 3rd generation dendrimers G 2,3 KL-(Leu)_4_ (D) and G 2,3 KL-diPalmitamide (D) were readily uptaken by cells compared to the control (cells treated with ON only) (Figure 3). To better understand the intracellular ONs distribution, cells were fixed and stained with DAPI as nuclear stain and fluorescence microscopy images indicated perinuclear delivery (Supplementary Figure S2).

For the rest of the study, OptiMEM^®^ medium was used for G 2,3 KL-(Leu)_4_ (D) formulation and HBG buffer was preferred for G 2,3 KL-diPalmitamide (D) formulation.

### 3.2. Cellular Uptake of Dendrimers/ON Complexes and Their Activity at Different Time-Points

**Figure 4:**
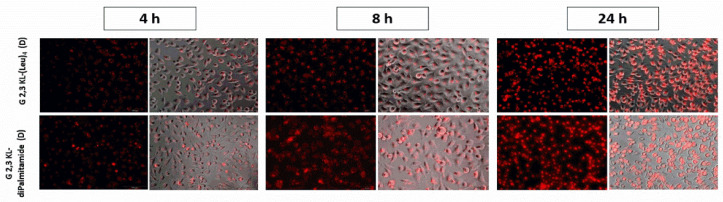
Cellular uptake as a measure of splice-correction levels of the lipophilic peptide dendrimers (G 2,3 KL-(Leu)_4_ (D) and G 2,3 KL-diPalmitamide (D)) at different time points. Dendrimers were formulated in OptiMEM^®^ and HBG, respectively, and complexed with Alexa-568-labelled ON before HeLa Luc/705 transfection in serum-containing media. Live cells were rinsed with DMEM^®^ with no phenol red (Invitrogen), before imaging with the fluorescence microscope (Olympus IX81). (magnification 20×, Scale bar = 100 µm).

**Figure 5:**
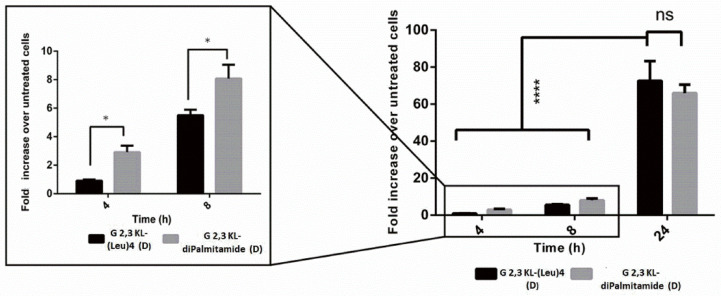
Luciferase activity as a measure of splice-correction levels of the lipophilic peptide dendrimers (G 2,3 KL-(Leu)_4_ (D) and G 2,3 KL-diPalmitamide (D)) at different time points. Dendrimers were formulated in OptiMEM^®^ and HBG, respectively. Luciferase activity levels were measured after HeLa Luc/705 transfection with the dendrimers in serum-containing media. Each bar represents the mean with the SEM of at least three independent experiments performed in triplicate (*n* ≥ 3). *p*-values were calculated by two-way ANOVA test, and the statistical difference was measured using post hoc Fisher’s LSD test. (ns: non-significant, * *p* ≤ 0.05, and **** *p* ≤ 0.0001).

Splice-switching activity and cellular uptake at different time points (4, 8, and 24 h) for the lipid-conjugated peptide dendrimers G 2,3 KL-(Leu)_4_ (D) and G 2,3 KL-diPalmitamide (D) were investigated. As shown in Figure 4, similar uptake behavior was noticed for both dendrimers at the corresponding time points. Meanwhile, for the activity, G 2,3 KL-diPalmitamide (D) shows slightly higher luciferase signals at early time points (Figure 5). This might imply a faster endosomal escape or nuclear entry rate compared to G 2,3 KL-(Leu)_4_ (D). The difference in the lipophilic moieties attached to the core (alkyl chain in G 2,3 KL-diPalmitamide (D) versus leucine residue in G 2,3 KL-(Leu)_4_ (D)) is a contributing factor and was previously demonstrated for siRNA [34].

### 3.3. Physical Characterization and Viability of Lipophilic Peptide Dendrimers

**Figure 6:**
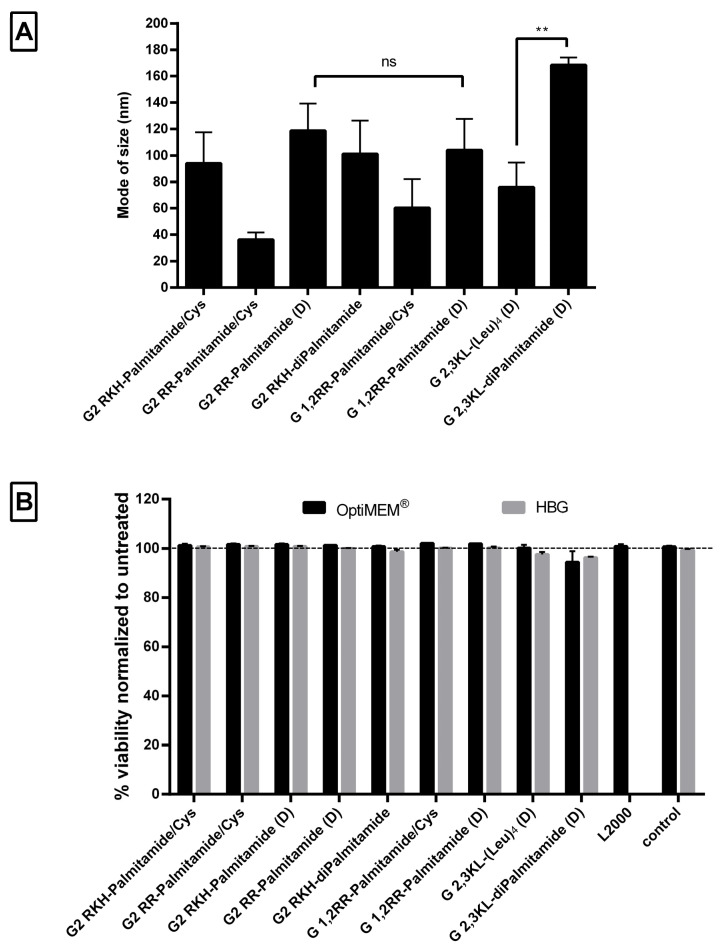
Size measurements of peptide dendrimer/Oligonucleotide complexes and the effect on cell viability. (**A**) Mode of size in (nm) of selected dendrimers. All complexes were formulated in OptiMEM^®^, except for G2 RKH-diPalmitamide and G 2,3 KL-diPalmitamide (D) which were formulated in HBG. (**B**) Percent viability of HeLa Luc/705 cells in comparison to untreated cells after transfection with dendrimer/ON complexes in serum-containing media, determined using the WST-1 assay. Values represent the mean with the SEM of at least three experiments performed in triplicate (*n* ≥ 3). Mode of size: most prevalent size in a sample. *p*-values were calculated by one-way ANOVA test and the statistical difference was measured using post hoc Fisher’s LSD test. (ns: non-significant, ** *p* ≤ 0.01).

Nanoparticle formation was confirmed using Nanoparticle Tracking Analysis (NTA), which showed that peptide dendrimer/ON complexes formed nanoparticles with a mode size (the most frequent size in the sample) of less than 250 nm at pH 7.4 (Figure 6A). The mean of size was around 155 ± 37 nm and the size distribution ≤100 nm (Supplementary Figure S3). The mode of size was preferred, since the mean size might be affected by aggregates and fails to represent the actual sizes in the sample [41,42]. Although there was no clear relation between the size and the activity, the size of the G 2,3 KL-diPalmitamide (D) complexes was significantly bigger than the other 3rd generation dendrimer G 2,3 KL-(Leu)_4_ (D). This is due to the higher complexation and tendency to aggregate for diPalmitamide [34].

Regarding the cellular viability, the WST-1 assay showed that none of the screened peptide dendrimers displayed any toxicity upon transfection in HeLa Luc/705 reporter cells in serum-containing media (Figure 6B).

### 3.4. Lipophilic Peptide Dendrimers Are Similar or Surpass the Transfection Efficiency of Lipofectamine (L2000) in Various Reporter Cells

**Figure 7:**
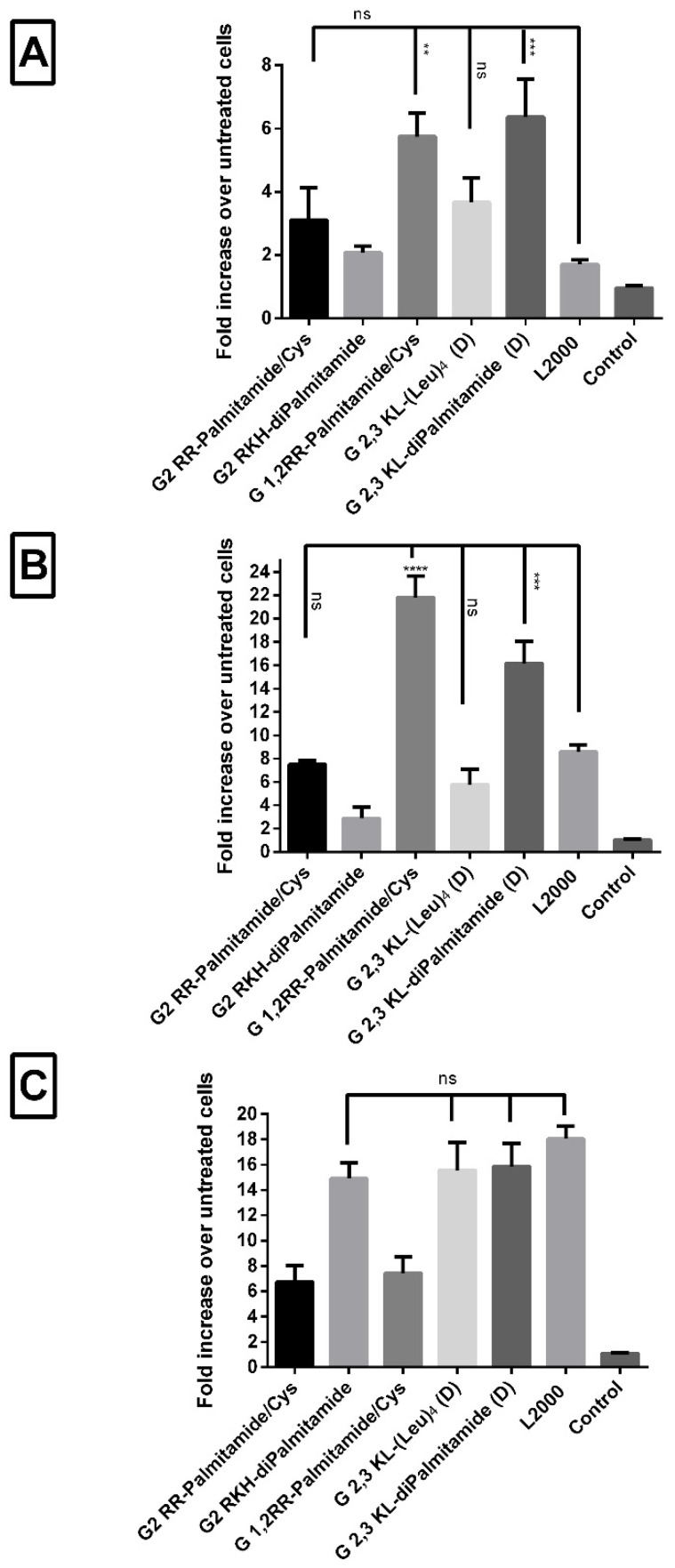
Transfection efficiency of peptide dendrimer/ON complexes in different cells. Comparisons of the fold increase in luciferase activity after transfection with selected 2nd and 3rd generation peptide dendrimers in serum-containing media in three different cells. (**A**) HuH7/705, (**B**) N2a/705, and (**C**) U-2 OS/705. All complexes were formulated in OptiMEM^®^ except for G2 RKH-diPalmitamide and G 2,3 KL-diPalmitamide (D) in HBG. ON complexed with L2000 was used as positive control and ON alone as negative control. ON concentration: 100 nM. Each bar represents the mean with the SEM of at least three experiments done in triplicate (*n* ≥ 3). *p*-values calculated by one-way ANOVA test, and statistical difference in the transfection efficiency between L2000 and peptide dendrimer/ON complexes was compared using post hoc Fisher’s LSD test (ns: non-significant, ** *p* ≤ 0.01, *** *p* ≤ 0.001 and **** *p* ≤ 0.0001).

Since different cells interact and behave differently upon treatment, it was of interest to test the transfection efficiency of the leading dendrimers in different reporter cells. Peptide dendrimer/ON complexes of selected 2nd and 3rd generation dendrimers were prepared as described previously in the text. Complexes were transfected into HuH7/705, Neuro 2a/705, and U-2 OS/705 cells to achieve a final concentration of 100 nM of ON. L2000 (gold-standard) was used as positive- and ON alone as negative control. In both HuH7/705 and Neuro 2a/705 cells, all the screened peptide dendrimers were as good as, or yielding significantly higher values, than L2000 (Figure 7A,B). Conversely, in U-2 OS/705 cells, only G2 RKH-diPalmitamide, G 2,3 KL-(Leu)_4_ (D), and G 2,3 KL-diPalmitamide (D) were as good as the gold standard L2000 (Figure 7C).

### 3.5. The Polymeric Excipient Polyvinylalcohol 18 Enhances Transfection Efficiency of Complexes in HeLa Luc/705 Reporter Cells in Serum-Containing Media

**Figure 8:**
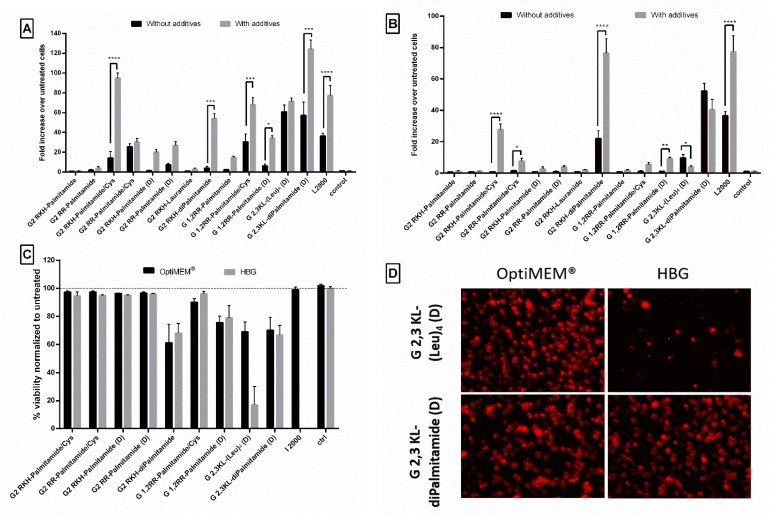
The effect of the polymeric excipient, PVA 18, on transfection efficiency and cell viability after transfection of HeLa Luc/705 cells with the lipophilic peptide dendrimer/ON complexes in serum-containing media. Fold increase in the luciferase activity (RLU normalized by the total amount of protein and related to values for untreated cells). (**A**) dendrimers were formulated in OptiMEM^®^, (**B**) dendrimers were formulated in HBG. ON complexed with L2000 was used as positive control and ON alone as negative control. ON concentration: 100 nM. (**C**) Viability of HeLa Luc/705 cells after transfection in serum-containing media with dendrimer/ON complexes with PVA18 excipient. Results were normalized to the viability of untreated cells. (**D**) Cellular uptake behavior after HeLa Luc/705 reporter cells transfection in serum-containing media of the 3rd generation peptide dendrimer/Alexa-568-labelled ON/PVA18 complexes formulated in different media. Complexes were incubated for 24 h, at 37 °C in a humidified incubator with 5% CO_2_. Live cells were rinsed with DMEM^®^ with no phenol red (Invitrogen), before imaging with the fluorescence microscope (Olympus IX81). (magnification 20×, Scale bar = 100 µm. Values represent the mean with the SEM of at least three experiments performed in triplicate (*n* ≥ 3). *p*-values calculated by Two-way ANOVA test and statistical difference with and without PVA 18 for each complex in each formulating buffer were compared using post hoc Fisher’s LSD test (ns: non-significant, * *p* ≤ 0.05, ** *p* ≤ 0.01, *** *p* ≤ 0.001 and **** *p* ≤ 0.0001).

Due to the unique properties and simple structure of the polymeric excipient Polyvinylalcohol 18, it is extensively used in biomedical and pharmaceutical applications [45]. It is also a good candidate as drug delivery system for small molecules and macromolecular gene therapeutics [46,47,48]. Previously, we demonstrated that transfection efficiency of peptide dendrimers/lipids can be further improved by adding different types of saccharides or polyol compounds [40]. Among those screened polymeric excipients, PVA18 was one of the best excipients to enhance transfection efficiency of peptide dendrimer/lipid hybrids [40]. In our current study, we observed that the addition of PVA18 to the lipophilic peptide dendrimers improved transfection efficiency significantly. The improvement was more pronounced for dendrimers that showed an initial activity (Figure 8). Once more, the polymeric excipient influences differently the same dendrimer when formulated in different buffers.

In OptiMEM^®^, PVA18 addition significantly increased the transfection efficiency of 2nd generation dendrimers: G2 RKH-Palmitamide/Cys, G2 RKH-diPalmitamide, G 1,2RR-Palmitamide/Cys, G 1,2RR-Palmitamide (D), and 3rd generation dendrimer: G 2,3 KL-diPalmitamide (D) up to 95-, 54-, 68-, 34-, and 124-fold over untreated, respectively (Figure 8A). For HBG, the increase in luciferase signals was observed in G2 RKH-Palmitamide/Cys and in G2 RKH-diPalmitamide formulations (28- and 76-fold, respectively) (Figure 8B). Except for G2 RKH-Palmitamide/Cys and G 1,2RR-Palmitamide (D), these values were significantly higher than formulations using L2000. Interestingly, it seems that the enhancing effect of the excipient is linked to the lipid component. We have earlier reported an enhancing effect of PVA18 using lipofectin, [40]. Hence, we tested the addition of PVA18 to lipofectamine and we observed a 2-fold increase in transfection efficiency in OptiMEM^®^. However, other buffers showed variable effects (Supplementary Figure S4) emphasizing the importance of the formulating media on the complexation of the dendrimers/ON/additives to achieve a potent transfection.

One explanation for the improvement in transfection after PVA18 addition, can be the ability of PVA18 to be adsorbed onto the surface of nanoparticles via H-bond formation [47]. This association impacts the nanoparticle interfacial properties, improves their stability, and reduces the risk of aggregation and/or coalescence, which is the case in using PVA 18 to improve the quality of nanoparticle complexes [48].

In regard to the effect of those lipophilic peptide dendrimer/ON complexes on the viability of the HeLa Luc/705 reporter cells, the addition of PVA18 to G 2,3 KL-(Leu)_4_ (D) and G 2,3 KL-diPalmitamide (D) showed variation from mild to severe toxicity. It was already reported by Orient et al. [49] that the conjugation of PVA with lipophilic moiety would not affect their compatibility with cells nor increase toxicity. However, we believe that the enhanced uptake effect exerted by the polymeric excipients on different complexes and the entry of more complexes inside the cells caused the toxicity. Interestingly, for the gold standard L2000, there was no toxicity observed for the concentration tested (Figure 8C). As hypothesized, the relatively loose adsorption of PVA onto the surface of nanoparticles at pH 7 is sufficient to improve cellular uptake [40]. However, adsorption is expected to become much stronger during the endosomal stage due to pH reduction to around 5.5 and would likely enhance the protective properties of PVA and facilitate endosomal escape.

Figure 8D presents the cellular uptake of dendrimer/ON/PVA18 complexes after 24 h of transfection. PVA18 seems to enhance cellular uptake for the 3rd generation peptide dendrimers. The lower signal of cells treated with G 2,3 KL-(Leu)_4_ (D) in HBG (almost no cells left) correlates with the low viability using the same buffer (Figure 8C).

### 3.6. Intracellular Distribution by Confocal Microscopy

Confocal microscopy permits collecting serial optical sections from thick specimens allowing to exactly detect the distribution inside a target [50]. It is known that fixation might alter the ON distribution [51,52]. Hence, we switched the protocol from fixation and the use of DAPI staining, into utilizing live confocal imaging to accurately investigate the distribution of the peptide dendrimers/ON within the cells with a better resolution.

**Figure 9:**
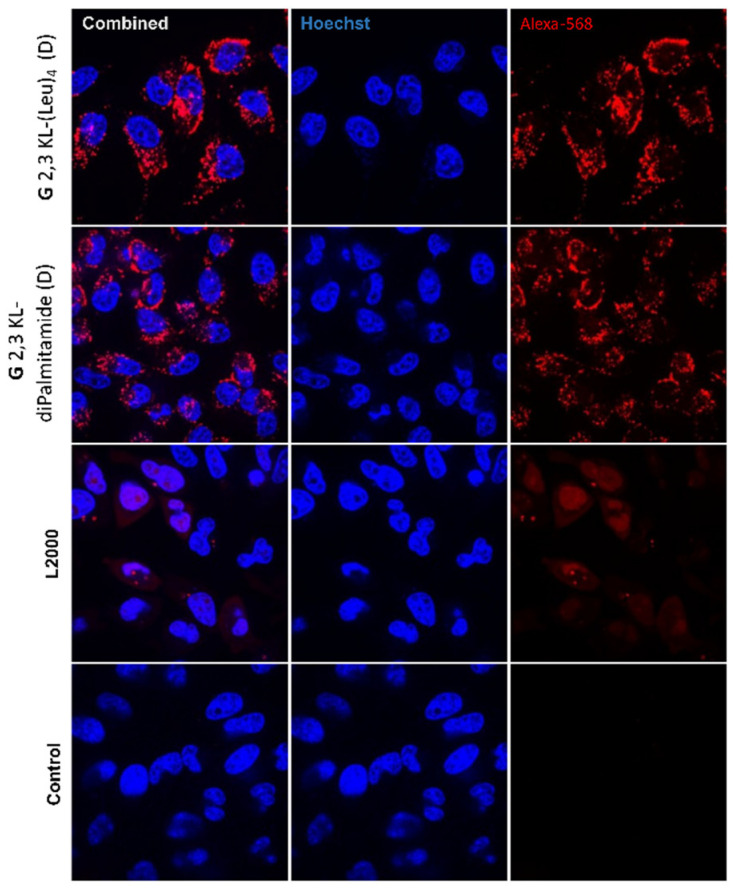
Live confocal microscopy imaging. Cellular uptake and distribution of ONs after HeLa Luc/705 reporter cell transfection in serum-containing media using the best performing 3rd generation peptide dendrimer/Alexa-568-labelled ON/complexes formulated in different media (G 2,3 KL-(Leu)_4_ (D) in OptiMEM^®^ and G 2,3 KL-diPalmitamide (D) in HBG). Cells were incubated for 24 h, at 37 °C in a humidified incubator with 5% CO_2_ before adding Hoechst dye (Thermofisher), following the manufacturer protocol. Live cell imaging was performed using a confocal microscope (A1R confocal, Nikon, Tokyo, Japan). Pictures were taken at a magnification 20×, and analyzed with NIS-Elements software (Nikon, Tokyo, Japan).

Hoechst dye highlighted the nucleus, whereas the ON was visualized through its conjugation to Alexa-568. The combined images showed the distribution of the ON within the cells (Figure 9). Confocal images indicated higher nuclear uptake in L2000 compared to the lipophilic peptide dendrimers. However, ON nuclear uptake does not always relate with activity; signals corresponding to nuclear uptake of ON within dendrimers could be too weak to be detected.

### 3.7. Effect of Different Buffers on Transfection Efficiency of the Lipophilic Peptide Dendrimers

Since buffer chemistry and composition affected the behavior of the lipophilic dendrimers, as mentioned, we next assessed their behavior in other buffers, such as DMEM^®^ and PBS.

**Figure 10:**
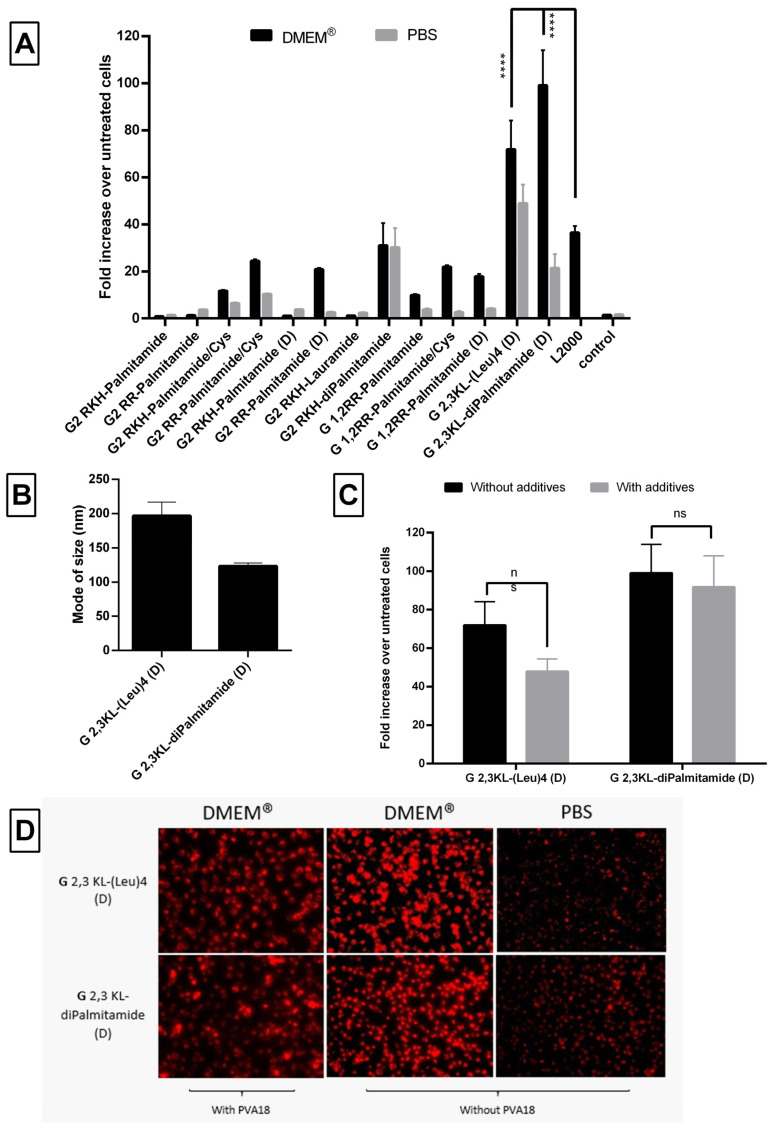
Evaluation of the behavior of the lipophilic peptide dendrimer/ON complexes. (**A**) Fold increase in luciferase signals after transfection of the HeLa Luc/705 cells in serum-containing media. (**B**) Mode of size (nm) of selected dendrimers formulated in DMEM^®^. (**C**) The effect on transfection of HeLa Luc/705 reporter cells in serum-containing media with lipophilic peptide dendrimer/ON complexes formulated in DMEM^®^ in presence of the polymeric excipient PVA 18. (**D**) Cellular uptake after transfection in serum-containing media of the selected 3rd generation peptide dendrimers/Alexa-568-labelled ON complexes in HeLa Luc/705 cells. Dendrimers were formulated with PVA18 in DMEM^®^ and without PVA18 in DMEM^®^ and PBS and incubated for 24 h, at 37 °C in a humidified incubator with 5% CO_2_. Live cells were rinsed with DMEM^®^ with no phenol red (Invitrogen), before imaging with the fluorescence microscope (Olympus IX81). (magnification 20×, Scale bar = 100 µm. Fold increase in luciferase activity: RLU normalized by the total amount of protein and related to values for untreated cells. Each bar represents the mean with the SEM of at least three independent experiments performed in triplicate (*n* ≥ 3). *p*-values were calculated by two-way ANOVA test and the statistical differences were measured using post hoc Fisher’s LSD test. (ns: non-significant, and **** *p* ≤ 0.0001).

Formulating G 2,3 KL-(Leu)_4_ (D) in DMEM^®^ increased the level of luciferase up to 72-fold and up to 99-fold for G 2,3 KL-diPalmitamide (D), while up to 49- and 21-fold for G 2,3 KL-(Leu)_4_ (D) and G 2,3 KL-diPalmitamide (D) when formulated in PBS, respectively, compared to untreated cells (Figure 10A). These results reemphasized the role of the formulating buffer and salt concentration on the configuration state of the dendrimers, thus affecting complexation, stability, and transfection ability [41,42]. The mode of size of the formed nanoparticles was around 200 nm for G 2,3 KL-(Leu)_4_ (D) and around 120 nm for G 2,3 KL-diPalmitamide (D) (Figure 10B). It seems that G 2,3 KL-diPalmitamide (D) complexes formulated in DMEM^®^ were smaller than those formulated in HBG (Figure 6A).

The effect of the polymeric additive PVA18 on the activity of the 3rd generation peptide dendrimers was also investigated when formulated in DMEM^®^. PVA18 did not cause any statistically significant change in luciferase signals for G 2,3 KL-(Leu)_4_ (D) and G 2,3 KL-diPalmitamide (D) (Figure 10C). However, cellular viability upon treatment with complexes formulated in DMEM^®^ with PVA18 was variable (Supplementary Figure S5).

Regarding uptake, we investigated the effect of different buffers (PBS and DMEM^®^) on the uptake of complexes of labeled ON. It was significant that complexes formed in PBS had much reduced cellular uptake, which might explain the low activity (Figure 10D). Additionally, we found a comparable cellular uptake of the 3rd generation peptide dendrimers, with or without, PVA18 in DMEM^®^ (Figure 10D).

## 4. Conclusions and Perspectives

In this report, 18 lipophilic peptide dendrimers were screened for their ability to transfect single strand splice-switching ONs. The 3rd generation peptide dendrimers G 2,3 KL-(Leu)_4_ (D) and G 2,3 KL-diPalmitamide (D) were found to be the best and most robust performing peptide dendrimers similar to what have been reported before with siRNA [34]. Both dendrimers act as a single component transfection vector with a better performance than the gold standard Lipofectamine L2000 in serum-containing media, and without significant toxicity. This study highlights the impact of the formulating medium, charge distribution, size, and type of amino acids in peptide dendrimers on the transfection efficiency. In addition, the significant capability of the polymeric PVA18 in enhancing transfection was noticed. Future work will focus on screening of additional lipophilic conjugates with different lengths and chemistries and evaluate their effect on the uptake and activity of the 2nd and 3rd generation dendrimers. The screening will also aim to thoroughly investigate the effects of the D-amino acid substitution. Based on the results of the screening, we will finally select the most effective and safe candidate to be tested in vivo.

## Data Availability

Not applicable.

## Supplementary Materials

The following are available online at https://www.mdpi.com/1999-4923/13/1/116/s1, Figure S1: Splice-switching of lipid conjugated Peptide Dendrimer/ON complexes delivered oligonucleotides is affected by stereochemistry of amino acids. Fold increase in luciferase signals after the transfection of the HeLa pLuc/705 cells in serum-containing media with lipid conjugated Peptide Dendrimer/ON complexes. Graphs represent the fold increase in RLUs normalized by the total amounts of protein of transfected versus untreated cells for the dendrimers complexed with ON at (N/P ratio = 20). ON complexes with L2000 were used as a positive control. ON concentration: 100 nM. Luciferase activity was analysed 24 h after transfection. Values represent the mean with the standard error of the mean (SEM) of at least three experiments performed in triplicate (*n* ≥ 3), Figure S2: Intracellular ONs distribution. Cellular uptake and ON intracellular distribution after transfection in serum-containing media with 3rd generation peptide dendrimers/ Alexa-568-labelled ON complexes in HeLa-705 reporter cells. Dendrimers were formulated in OptiMEM^®^ (G 2,3 KL-(Leu)_4_ (D)) or HBG (G 2,3 KL-dipalmitamide (D)) and incubated for 24 h, at 37 °C in a humidified incubator with 5% CO_2_. Before imaging using fluorescence microscopy, cells were fixed using iced methanol, then treated with DAPI stain for 15 min, (magnification 20×, Scale bar = 100 μm), Figure S3: Size measurement. Size measurements for the formulated lipid conjugated peptide dendrimer/Oligonucleotide complexes. (**a**) Mean of size and (**b**) size distribution in (nm) of selected dendrimers formulated in the appropriate buffers. All were formulated in OptiMEM^®,^ except for G2 RKH-diPalmitamide and G 2,3 KL-dipalmitamide in HBG. The pH of all the formulating buffers is between [7.2–7.4]. Values represent the mean with the standard error of the mean (SEM) of at least three experiments performed in triplicate (*n* ≥ 3). Size distribution (SD): a measure of the polydispersity of the sample. P-values were calculated by one-way ANOVA test and the statistical differences was measured using post hoc Fisher’s LSD test. (ns: non-significant), Figure S4.: Splice-switching of the gold standard L2000/ON complexes delivered oligonucleotides with and without PVA18.Fold increase in luciferase signals after the transfection of the HeLa pLuc/705 cells in serum-containing media with L2000/ON complexes formulated in OptiMEM^®^, HBG and DMEM^®^. Graphs represent the fold increase in RLUs normalized by the total amounts of protein of transfected versus untreated cells. ON concentration: 100 nM. Luciferase activity was analysed 24 h after transfection. Each bar represents the mean with the standard error of the mean (SEM) of at least three independent experiments performed in triplicate (*n* ≥ 3). P-values were calculated by two-way ANOVA test and the statistical differences was measured using post hoc Fisher’s LSD test. (ns: non-significant and **** *p* ≤ 0.0001), Figure S5: % Viability after transfection of complexes formulated in DMEM^®^ and supplemented with Polyvinylalcohol 18 (PVA 18). Viability of HeLa Luc/705 cells after transfection under serum condition with dendrimer/ON complexes formulated in DMEM^®^ with PVA18 excipient. ON complexed with L2000 were used as positive control and ON alone as negative control. ON concentration: 100 nM. Results were normalized to the level of untreated cells. Each bar represents the mean with the standard error of the mean (SEM) of at least three independent experiments performed in triplicate (*n* ≥ 3), Table S1: Evaluation of Peptide Dendrimers for Splice-Switching Oligonucleotide Transfection; Table S2: Evaluation of Peptide Dendrimers Complexed with Polyvinylalcohol 18 (PVA18) for Splice-Switching Oligonucleotide Transfection; Table S3: Synthesis of peptide dendrimers, Figure S6: RKH-Plamitamide (RKH)4(KLL)2KKC16, it was obtained after manual synthesis as foamy colorless solid after preparative RP-HPLC (20.5 mg, 9%). (b) Analytical RP-HPLC: tr = 3.73 min (100% A to 100% D in 7.5 min, λ = 214 nm). (c), (d) MS (ESI+): C136H249N49O21 calc./obs. 2904.99/2904.99; Figure S7: (a) Structure of G2 RR-Palmitamide (RR)4(KLL)2KKC16, it was obtained after manual synthesis as foamy colorless solid after preparative RP-HPLC (7 mg, 3.5%). (b) Analytical RP-HPLC: tr = 3.99 min (100% A to 100% D in 7.5 min, λ = 214 nm). (c,d) MS (ESI+): C112H221N45O17 calc./obs. 2468.78/2468.78; Figure S8: RKH-Plamitamide/Cys (RKH)_4_(KLL)_2_KKC16-C, it was obtained after manual synthesis as foamy colorless solid after preparative RP-HPLC (15.5 mg, 7%). (**b**) Analytical RP-HPLC: tr = 3.85 min (100% A to 100% D in 7.5 min, λ= 214 nm). (**c**,**d**) MS (ESI+): C_139_H_254_N_50_O_22_S calc./obs. 3008.00/3008.00; Figure S9: (a) Structure of G2 RR-Palmitamide/Cys (RR)4(KLL)2KKC16-C, it was obtained after manual synthesis as foamy colorless solid after preparative RP-HPLC (29.4 mg, 14%). (b) Analytical RP-HPLC: tr = 4.05 min (100% A to 100% D in 7.5 min, λ = 214 nm). (c,d) MS (ESI+): C115H226N46O18S calc./obs. 2571.79/2571.79; Figure S10: (a) Structure of G2 RKH-Palmitamide (D) (rkh)4(kll)2kkC16, it was obtained after manual synthesis as foamy colorless solid after preparative RP-HPLC (27.8 mg, 13%). (b) Analytical RP-HPLC: tr = 3.65 min (100% A to 100% D in 7.5 min, λ = 214 nm). (c,d) MS (ESI+): C136H249N49O21 calc./obs. 2904.99/2904.99; Figure S11: (**a**) Structure of G2 RR-Palmitamide (D) (rr)_4_(kll)_2_kkC16, it was obtained after manual synthesis as foamy colorless solid after preparative RP-HPLC (39.8 mg, 23%). (**b**) Analytical RP-HPLC: tr = 4.02 min (100% A to 100% D in 7.5 min, λ = 214 nm). (**c**,**d**) MS (ESI+): C_112_H_221_N_45_O_17_ calc./obs. 2468.78/2468.77; Figure S12: (**a**) Structure of G2 RKH-Lauramide (RKH)_4_(KLL)_2_KKC12, it was obtained after manual synthesis as foamy colorless solid after preparative RP-HPLC (12.5 mg, 5%). (**b**) Analytical RP-HPLC: tr = 3.20 min (100% A to 100% D in 7.5 min, λ = 214 nm). (**c**,**d**) MS (ESI+): C_132_H_241_N_49_O_21_ calc./obs. 2848.93/2848.91; Figure S13: (a) Structure of G2 RKH-diPalmitamide (RKH)4(KLL)2KKC16-KC16, it was obtained after manual synthesis as foamy colorless solid after preparative RP-HPLC (12.2 mg, 5%). (b) Analytical RP-HPLC: tr = 5.18 min (100% A to 100% D in 7.5 min, λ = 214 nm). (c,d) MS (ESI+): C158H291N51O23 calc./obs. 3271.32/3271.32; Figure S14: (**a**) Structure of G1,2 RR-Palimitamide (RR)_4_(KRR)_2_KKC16, it was obtained after manual synthesis as foamy colorless solid after preparative RP-HPLC (21.7 mg, 10%). (**b**) Analytical RP-HPLC: tr = 3.75 min (100% A to 100% D in 7.5 min, λ = 214 nm). (**c**,**d**) MS (ESI+): C_112_H_225_N_57_O_17_ calc./obs. 2640.85/2640.85; Figure S15: (**a**) Structure of G1,2 RR-Palmitamide/Cys (RR)_4_(KRR)_2_KKC16-C, it was obtained after manual synthesis as foamy colorless solid after preparative RP-HPLC (24.4 mg, 10%). (**b**) Analytical RP-HPLC: tr = 3.75 min (100% A to 100% D in 7.5 min, λ = 214 nm). (**c**,**d**) MS (ESI+): C_115_H_230_N_58_O_18_S calc./obs. 2743.86/2743.86; Figure S16: (a) Structure of G1,2 RR-Palmitamide (D) (rr)4(krr)2kkC16, it was obtained after manual synthesis as foamy colorless solid after preparative RP-HPLC (38.2 mg, 16%). (b) Analytical RP-HPLC: tr = 3.77 min (100% A to 100% D in 7.5 min, λ = 214 nm). (c), (d) MS (ESI+): C112H225N57O17 calc./obs. 2640.85/2640.85; Figure S17: (**a**) Structure of G2,3 KL (D) (kl)_8_(kkl)_4_(kll)_2_kgsc, it was obtained after manual synthesis as foamy colorless solid after preparative RP-HPLC (15.3 mg, 5%). (**b**) Analytical RP-HPLC: tr = 2.91 min (100% A to 100% D in 7.5 min, λ = 214 nm). (**c**,**d**) MS (ESI+): C_218_H_420_N_58_O_39_S calc./obs. 4507.24/4507.24; Figure S18: (a) Structure of G2,3 KL-no core (D) (kl)8(kkl)4(kll)2k, it was obtained after manual synthesis as foamy colorless solid after preparative RP-HPLC (18.2 mg, 5%). (b) Analytical RP-HPLC: tr = 2.89 min (100% A to 100% D in 7.5 min, λ = 214 nm). (c,d) MS (ESI+): C210H407N55O35 calc./obs. 4260.18/4260.18.

## Abbreviations

ANOVA	Analysis of Variance
CO_2_	Carbon Dioxide
DAPI	4′,6-diamidino-2-phenylindole
DMEM	Dulbecco’s modified Eagle’s medium
DNA	Deoxyribonucleic Acid
DOPE	Dioleoyl-phosphatidylethanolamine
DOTMA	1,2-di-O-octadecenyl-3-trimethylammonium propane
FBS	Fetal bovine serum
HBG	HEPES Buffered Glucose
HEPES	4-(2-hydroxyethyl)-1-piperazineethanesulfonic acid
HPLC	High Performance Liquid Chromatography
L2000	Lipofectamine 2000
LSD	Least Significant Difference
N/P ratio	Nitrogen/Phosphate ratio
NTA	Nanoparticles Tracking Analysis
ON	Oligonucleotide
OptiMEM^®^	Reduced-Serum Medium is an improved Minimal Essential Medium (MEM)
PAMAM	Polyamidoamine
PBS	Phosphate Buffered Saline
PEI	Polyethyleneimine
PEO	polyethylene oxide
PPI	Polypropyleneimine
PVA	Polyvinylalcohol
RLU	Relative Light Unit
SEM	Standard error of the mean
SiRNA	Small interfering RNA
SPPS	Solid-phase peptide synthesis
WST-1	(2-(4-Iodophenyl)-3-(4-nitrophenyl)-5-(2,4-disulfophenyl)-2H-tetrazolium sodium salt
λem	Emission wavelength
λex	Excitation wavelength
