# Supplementary material for: Lipophilic Peptide Dendrimers for Delivery of Splice-Switching Oligonucleotides
Source: Pharmaceutics. 2021 Jan 18;13(1):116. doi: 10.3390/pharmaceutics13010116 (PMC7831936; doi:10.3390/pharmaceutics13010116)
Supplement: Supplementary file 1 [file pharmaceutics-13-00116-s001.pdf]

pharmaceutics

Supplementary Materials: Lipophilic Peptide Dendrimers for Delivery of Splice-Switching Oligonucleotides

Haneen Daralnakhla, Osama Saher, Susanna Zamolo, Safa Bazaz, Jeremy P. Bost, Marc Heitz, Karin E. Lundin, Samir EL Andaloussi, Tamis Darbre, Jean-Louis Reymond, Rula Zain and C. I. Edvard Smith

Part 1: Figures and Tables

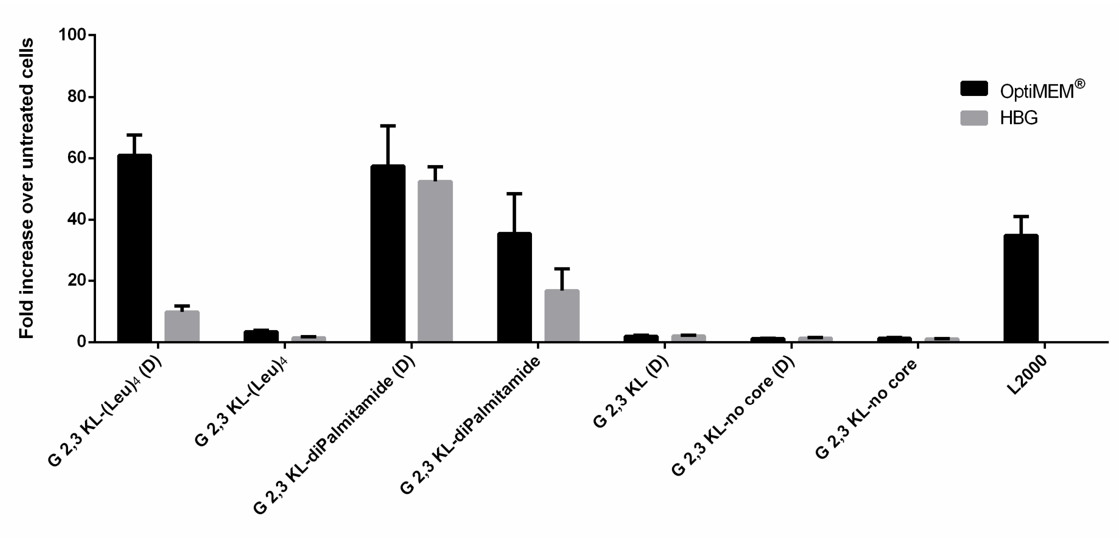

Figure S1. Splice-switching of lipid conjugated Peptide Dendrimer/ON complexes delivered oligonucleotides is affected by stereochemistry of amino acids. Fold increase in luciferase signals after the transfection of the HeLa pLuc/705 cells in serum-containing media with lipid conjugated Peptide Dendrimer/ON complexes. Graphs represent the fold increase in RLUs normalized by the total amounts of protein of transfected versus untreated cells for the dendrimers complexed with ON at (N/P ratio = 20). ON complexes with L2000 were used as a positive control. ON concentration: 100 nM. Luciferase activity was analysed 24 h after transfection. Values represent the mean with the standard error of the mean (SEM) of at least three experiments performed in triplicate ($n \geq 3$).

G 2,3 KL-(Leu)$_4$ (D)

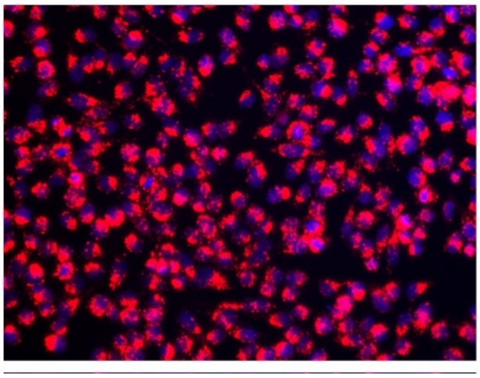

G 2,3 KL-dipalmitamide (D)

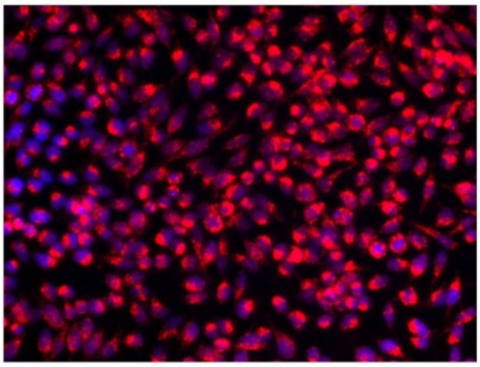

Figure S2. Intracellular ONs distribution. Cellular uptake and ON intracellular distribution after transfection in serum-containing media with 3rd generation peptide dendrimers/ Alexa-568-labelled ON complexes in HeLa-705 reporter cells. Dendrimers were formulated in OptiMEM® (G 2,3 KL-(Leu)$_4$ (D)) or HBG (G 2,3 KL-dipalmitamide (D)) and incubated for 24 h, at 37 °C in a humidified incubator with 5% CO_2. Before imaging using fluorescence microscopy, cells were fixed using iced methanol, then treated with DAPI stain for 15 min, (magnification 20×, Scale bar = 100 μm).

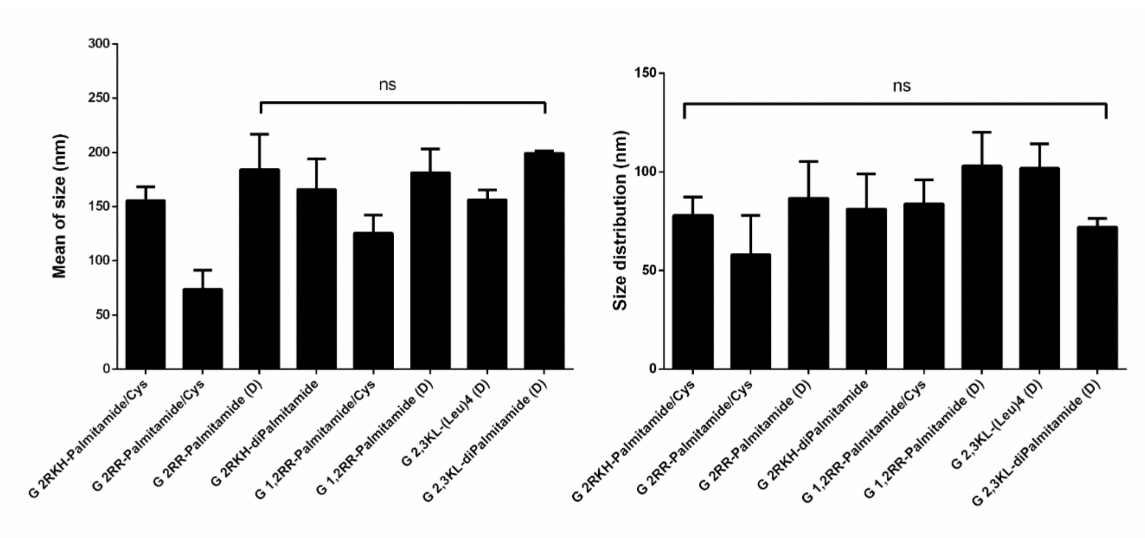

(**a**) (**b**)

Figure S3. Size measurement. Size measurements for the formulated lipid conjugated peptide dendrimer/Oligonucleotide complexes. (**a**) Mean of size and (**b**) size distribution in (nm) of selected dendrimers formulated in the appropriate buffers. All were formulated in OptiMEM®, except for G2 RKH-diPalmitamide and G 2,3 KL-dipalmitamide in HBG. The pH of all the formulating buffers is between [7.2–7.4]. Values represent the mean with the standard error of the mean (SEM) of at least three experiments performed in triplicate ($n \geq 3$). Size distribution (SD): a measure of the polydispersity of the sample. P-values were calculated by one-way ANOVA test and the statistical differences was measured using post hoc Fisher's LSD test. (ns: non-significant).

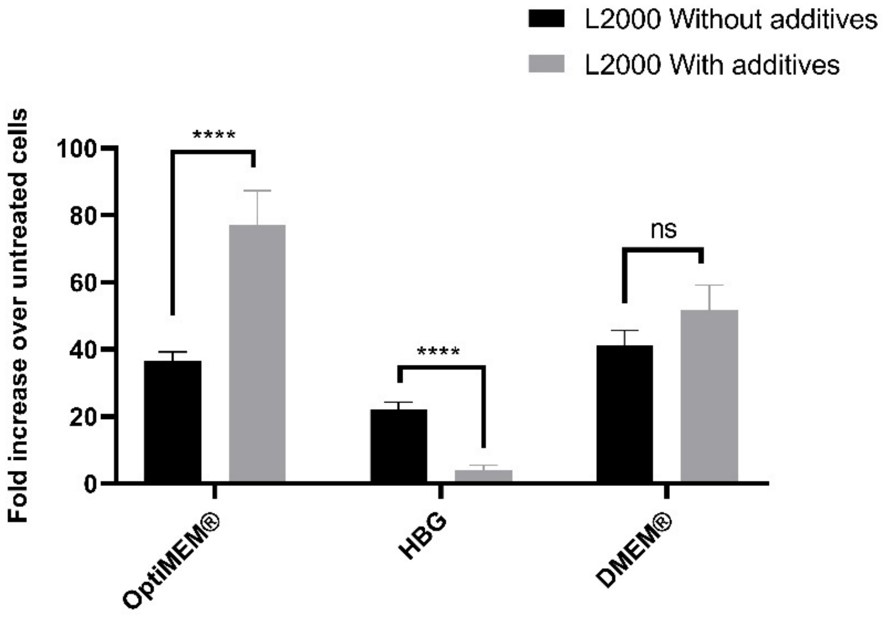

Figure S4. Splice-switching of the gold standard L2000/ON complexes delivered oligonucleotides with and without PVA18.Fold increase in luciferase signals after the transfection of the HeLa pLuc/705 cells in serum-containing media with L2000/ON complexes formulated in OptiMEM®, HBG and DMEM®. Graphs represent the fold increase in RLUs normalized by the total amounts of protein of transfected versus untreated cells. ON concentration: 100 nM. Luciferase activity was analysed 24 h after transfection. Each bar represents the mean with the standard error of the mean (SEM) of at least three independent experiments performed in triplicate ($n \geq 3$). P-values were calculated by two-way ANOVA test and the statistical differences was measured using post hoc Fisher's LSD test. (ns: non-significant and **** $p \leq 0.0001$).

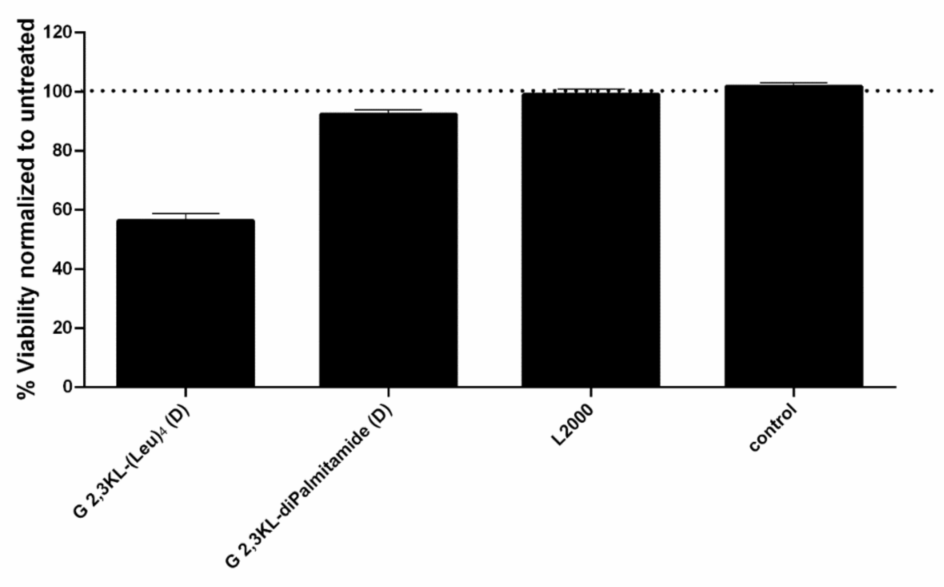

Figure S5. % Viability after transfection of complexes formulated in DMEM® and supplemented with Polyvinylalcohol 18 (PVA 18). Viability of HeLa Luc/705 cells after transfection under serum condition with dendrimer/ON complexes formulated in DMEM® with PVA18 excipient. ON complexed with L2000 were used as positive control and ON alone as negative control. ON concentration: 100 nM. Results were normalized to the level of untreated cells. Each bar represents the mean with the standard error of the mean (SEM) of at least three independent experiments performed in triplicate ($n \geq 3$).

Table S1. Evaluation of Peptide Dendrimers for Splice-Switching Oligoneclutide Transfection.

Code	Activity (%)				Viability(%)		Size (nm)		
	OptiMEM®	HBG	DMEM®	PBS	OptiMEM®	HBG	Mean	Mode	Standard deviation
**2nd Generation Peptide Dendrimers**									
G2 RKH-Palmitamide	0.83 ± 0.24	0.74 ± 0.21	0.89 ± 0.28	1.41 ± 0.70	N/A	N/A	N/A	N/A	N/A
G2 RR-Palmitamide	1.89 ± 0.55	0.87 ± 0.25	1.31 ± 0.41	3.78 ± 1.89	N/A	N/A	N/A	N/A	N/A
G2 RKH-Plamitamide/Cys	14.14 ± 4	0.95 ± 0.27	11.75 ± 3.72	6.50 ± 3.25	101.15 ± 58.40	100.60 ± 58.08	155.33 ± 63.41	94.00 ± 38.38	78.00 ± 31.84
G2 RR-Palmitamide/Cys	25.61 ± 7.3	1.52 ± 0.44	24.52 ± 7.75	10.37 ± 5.19	101.54 ± 58.63	100.78 ± 58.19	73.75 ± 30.11	36.25 ± 14.80	58.00 ± 23.68
G2 RKH-Palmitamide (D)	1.63 ± 0.47	0.95 ± 0.27	1.15 ± 0.36	3.81 ± 1.91	101.54 ± 58.63	100.78 ± 58.19	N/A	N/A	N/A
G2 RR-Palmitamide (D)	7.37 ± 2.1	1.02 ± 0.30	20.93 ± 6.62	2.60 ± 1.30	101.25 ± 58.46	100.01 ± 57.74	184.33 ± 75.25	118.83 ± 48.51	86.67 ± 35.38
G2 RKH-Lauramide	1.05 ± 0.3	0.96 ± 0.28	1.21 ± 0.38	2.41 ± 1.20	N/A	N/A	N/A	N/A	N/A
G2 RKH-diPalmitamide	4.03 ± 1.1	22.15 ±6.39	30.99 ± 9.80	30.25 ± 15.13	100.68 ± 58.13	98.64 ± 56.95	165.60 ± 67.61	101.20 ± 41.31	81.20 ± 33.15
G 1,2 RR-Palmitamide	2.40 ± 0.69	0.96 ± 0.28	9.95 ± 3.15	3.79 ± 1.90	N/A	N/A	N/A	N/A	N/A
G1,2 RR-Palmitamide/Cys	30.64 ±8.8	1.19 ± 0.34	21.92 ± 6.93	2.62 ± 1.31	101.99 ± 58.89	100.16 ± 57.83	125.50 ± 51.24	60.25 ± 24.60	83.75 ± 34.19
G1,2 RR-Palmitamide (D)	6.17 ± 1.7	1.36 ± 0.39	17.81 ± 5.63	4.08 ± 2.04	101.84 ± 58.80	100.27 ± 57.89	181.25 ± 74.00	104.00 ± 42.46	103.00 ± 42.05
**3rd Generation Peptide Dendrimers**									
G2,3 KL (D)	1.85 ± 0.54	1.98 ± 0.57	N/A	N/A	N/A	N/A	N/A	N/A	N/A
G2,3 KL-no core (D)	1.12 ±0.35	1.32 ± 0.38	N/A	N/A	N/A	N/A	N/A	N/A	N/A
G2,3 KL no core	1.28 ± 0.37	1.06 ± 0.31	N/A	N/A	N/A	N/A	N/A	N/A	N/A
G2,3 KL-(Leu)4	3.42 ± 0.99	1.46 ± 0.42	N/A	N/A	N/A	N/A	N/A	N/A	N/A
G2,3 KL- diPalmitamide	35.45 ± 10.2	16.74 ±4.83	N/A	N/A	N/A	N/A	N/A	N/A	N/A
G2,3 KL-(Leu)4 (D)	60.95 ±17.6	9.86 ± 2.85	71.93 ± 22.75	48.91± 24.46	100.21 ± 57.85	97.53 ± 56.31	156.25 ± 63.79 (135.00 ± 55.11)*	76.00±31.03 (196.75 ± 80.32)*	101.75 ± 41.54 (70.00 ± 28.58)*
G2,3 KL- diPalmitamide (D)	57.42 ± 16.6	52.43 ±15.13	99.08 ± 31.33	21.43 ± 10.72	94.40 ± 54.50	96.20 ± 55.54	199.25 ± 81.34 (152.50 ± 62.26)*	168.50 ± 68.79 (123.50 ± 50.42)*	72.00 ± 29.39 (66.50 ± 27.15)*
L2000	38.30 ± 11.06	20.45 ± 5.90	39.35 ± 11.36	N/A	100.77 ± 58.18	N/A	N/A	N/A	N/A
Control	1.29 ± 37	1.12 ± 0.32	1.44 ± 0.46	1.61 ± 0.80	100.63 ± 58.10	99.38 ± 57.38	N/A	N/A	N/A

*For the best formulating dendrimers G2,3 KL-$(Leu)_4$ (D) and G2,3 KL-diPalmitamide (D), numbers between prackets are the values after formulating them in DMEM. Abbreviations: OptiMEM®: Reduced-Serum Medium is an improved Minimal Essential Medium (MEM); HBG: HEPES Buffered Glucose;

DMEM®: Dulbecco's modified Eagle's medium; PBS: Phosphate Buffered Saline; L2000: Lipofectamine; R: Arginine; K: Lysine; H: Histidine; L: Leucine; Leu: Leucine; G: generation; D: D stereochemistry; nm: nanometer; N/A: not available.

Table S2. Evaluation of Peptide Dendrimers Complexed with Polyvinylalcohol 18 (PVA18) for Splice-Switching Oligonucleotide Transfection.

**Code**	**Activity (%)**				**Viability (%)**		
	**OptiMEM®**	**HBG**	**DMEM®**	**PBS**	**OptiMEM®**	**HBG**	**DMEM®**
**2nd Generation Peptide Dendrimers**							
G2 RKH-Palmitamide	1.14 ± 0.46	1.28 ± 0.48	N/A	N/A	N/A	N/A	N/A
G2 RR-Palmitamide	4.01 ± 1.64	1.15 ± 0.44	N/A	N/A	N/A	N/A	N/A
G2 RKH-Plamitamide/Cys	94.86 ± 38.73	27.75 ± 10.49	N/A	N/A	97.42 ± 43.57	94.57 ± 42.29	N/A
G2 RR-Palmitamide/Cys	30.66 ± 12.52	7.74 ± 2.93	N/A	N/A	97.42 ± 43.57	94.82 ± 42.41	N/A
G2 RKH-Palmitamide (D)	20.11 ± 8.21	2.73 ± 1.03	N/A	N/A	96.38 ± 43.10	94.82 ± 42.41	N/A
G2 RR-Palmitamide (D)	26.89 ± 10.98	3.86 ± 1.46	N/A	N/A	96.77 ± 43.28	95.97 ± 42.92	N/A
G2 RKH-Lauramide	3.11 ± 1.27	2.07 ± 0.78	N/A	N/A	N/A	N/A	N/A
G2 RKH-diPalmitamide	54.09 ± 22.08	76.41 ± 28.88	N/A	N/A	78.06 ± 34.91	68.76 ± 30.75	N/A
G1,2 RR-Palmitamide	14.84 ± 6.06	1.79 ± 0.68	N/A	N/A	N/A	N/A	N/A
G1,2 RR-Palmitamide/Cys	68.08 ± 27.79	5.58 ± 2.11	N/A	N/A	90.18 ± 40.33	96.23 ± 43.03	N/A
G1,2 RR-Palmitamide (D)	34.26 ± 13.99	9.26 ± 3.50	N/A	N/A	75.56 ± 33.79	78.94 ± 35.30	N/A
**3rd Generation Peptide Dendrimers**							
G2,3 KL (D)	N/A	N/A	N/A	N/A	N/A	N/A	N/A
G2,3 KL-no core (D)	N/A	N/A	N/A	N/A	N/A	N/A	N/A
G2,3 KL no core	N/A	N/A	N/A	N/A	N/A	N/A	N/A
G2,3 KL-(Leu)4	N/A	N/A	N/A	N/A	N/A	N/A	N/A
G2,3 KL- diPalmitamide	N/A	N/A	N/A	N/A	N/A	N/A	N/A
G2,3 KL-(Leu)4 (D)	71.39 ± 29.15	3.79 ± 1.43	47.88 ± 16.93	N/A	67.91 ± 30.37	24.12 ± 10.78	56.40 ± 32.56
G2,3 KL- diPalmitamide (D)	124.20 ± 50.70	40.32 ± 15.24	91.82 ± 32.46	N/A	66.35 ± 29.67	63.01 ± 28.18	92.37 ± 53.33
L2000	65.23 ± 26.63	7.55 ± 3.08	47.97 ± 19.58	N/A	100.74 ± 45.05	100.56 ± 45.12	100.21 ± 41.87
Control	1.23 ± 0.35	1.09 ± 0.41	1.07 ± 0.21	N/A	101.86 ± 45.55	99.94 ± 44.69	100.58 ± 43.33

Abbreviations: OptiMEM®: Reduced-Serum Medium is an improved Minimal Essential Medium (MEM); HBG: HEPES Buffered Glucose; DMEM®: Dulbecco's modified Eagle's medium; PBS: Phosphate Buffered Saline; L2000: Lipofectamine; R: Arginine; K: Lysine; H: Histidine; L: Leucine; Leu: Leucine; G: generation; D: D stereochemistry; nm: nanometer; N/A: not available.

Part 2: Synthesis and Characterization

1. Materials and reagents

All reagents, salts and buffers were used as purchased from Sigma Aldrich (Buch, Switzerland), Fluorochem Ltd (Hadfield, United Kingdom), Iris Biotech Gmbh (Marktredwitz, Germany), TCI (Tokyo Chemical Company) (Eschborn, Germany), GL Biochem (Shanghai, China). Amino acids were used as the following derivatives: Fmoc-Ser(tBu)-OH, Fmoc-Gly-OH, Fmoc-Cys(Trt)-OH, Fmoc-Leu-OH, Fmoc-D-Leu-OH, Fmoc-Lys(Boc)-OH, Fmoc-D-Lys(Boc)-OH, Fmoc-Lys(Fmoc)-OH, Fmoc-D-Lys(Fmoc)-OH, Fmoc-Lys(Alloc)-OH, Fmoc-D-Lys(Alloc)-OH, Fmoc-Arg(Pbf)-OH and Fmoc-His(Trt)-OH were purchased by Iris Biotech GmbH (Marktredwitz, Germany), or GL Biochem (Shanghai, China). Rink Amide AM resin LL was purchased from Novabiochem (Darmstadt, Germany). Peptide dendrimers synthesis was performed manually in polypropylene syringes fitted with a polyethylene frit, a Teflon stopcock and stopper or automatically by CEM Liberty Blue Automated Microwave Peptide Synthesizer (Kamp-Lintfort, Germany). Analytical RP-HPLC was performed with an Ultimate 3000 Rapid Separation LC-MS System (DAD-3000RS diode array detector) using an Acclaim RSLC 120 C18 column (2.2 μm, 120 Å, 3 × 50 mm, flow 1.2 mL/min) from Dionex (Reinach, Switzerland). Data recording and processing was done with Dionex Chromeleon Management System Version 6.80 (analytical RP-HPLC). All RP-HPLC were using HPLC-grade acetonitrile and Milli-Q deionized water. The elution solutions were: A Milli-Q deionized water containing 0.05% TFA; D Milli-Q deionized water/acetonitrile (10:90, *v*/*v*) containing 0.05% TFA. Preparative RP-HPLC was performed with a Waters automatic Prep LC Controller System containing the four following modules: Waters2489 UV/Vis detector, Waters2545 pump, Waters Fraction Collector III and Waters 2707 Autosampler. A Dr. Maisch GmbH Reprospher column (C18-DE, 100 × 30 mm, particle size 5 μm, pore size 100 Å, flow rate 40 mL/min) was used. Compounds were detected by UV absorption at 214 nm using a Waters 248 Tunable Absorbance Detector. Data recording and processing was performed with Waters ChromScope version 1.40 from Waters Corporation. All RP-HPLC were using HPLC-grade acetonitrile and Milli-Q deionized water. The elution solutions were: A: Milli-Q deionized water containing 0.1% TFA; D: Milli-Q deionized water/acetonitrile (10:90, *v*/*v*) containing 0.1% TFA. MS spectra were recorded on a Thermo Scientific LTQ OrbitrapXL. MS spectra were provided by the MS analytical service of the S4 Department of Chemistry and Biochemistry at the University of Bern (group PD Dr. Stefan Schürch).

2. Peptide Dendrimers Library

Table S3. Synthesis of peptide dendrimers.

Code	Sequence [a)]	Yield [b)] mg (%)	MS [c)] calc/obs
**2nd Generation Peptide Dendrimers**			
G2 RKH-Palmitamide	$(RKH)_4(KLL)_2KKC16$	20.5 (9)	2904.99/2904.99
G2 RR-Palmitamide	$(RR)_4(KLL)_2KKC16$	7 (3)	2468.78/2468.78
G2 RKH-Plamitamide/Cys	$(RKH)_4(KLL)_2KKC16$-C	15.5 (7)	3008.00/3008.00
G2 RR-Palmitamide/Cys	$(RR)_4(KLL)_2KKC16$-C	29.4 (14)	2571.79/ 2571.79
G2 RKH-Palmitamide (D)	$(rkh)_4(kll)_2kkC16$	27.8 (13)	2904.99/2904.99
G2 RR-Palmitamide (D)	$(rr)_4(kll)_2kkC16$	39.8 (23)	2848.93/2848.91
G2 RKH-Lauramide	$(RKH)_4(KLL)_2KKC12$	12.5 (5)	2468.78/2468.77
G2 RKH-diPalmitamide	$(RKH)_4(KLL)_2KKC16$-KC16	12.2 (5)	3271.32/3271.32
G 1,2 RR-Palmitamide	$(RR)_4(KRR)_2KKC16$	21.7 (10)	2640.85/2640.85
G1,2 RR-Palmitamide/Cys	$(RR)_4(KRR)_2KKC16$-C	24.4 (10)	2743.86/2743.86
G1,2 RR-Palmitamide (D)	$(rr)_4(krr)_2kkC16$	38.2 (16)	2640.85/2640.85
**3rd Generation Peptide Dendrimers**			
G2,3 KL (D)	$(kl)_8(kkl)_4(kll)_2kgsc$	15.3 (5)	4507.24/4507.24
G2,3 KL-no core (D)	$(kl)_8(kkl)_4(kll)_2k$	18.2 (5)	4260.18/4260.18
G2,3 KL no core [d)]-MH30	$(KL)_8(KKL)_4(KLL)_2K$	37.7 (8)	4260.18/4260.19
G2,3 KL-(Leu)4 [d)]-MH18	$(KL)_8(KKL)_4(KLL)_2KLLLL$	52.7 (8)	4712.51/4712.52
G2,3 KL-diPalmitamide [d)]-MH13	$(KL)_8(KKL)_4(KLL)_2KKC16KC16$	26.8 (4)	4992.83/4992.82
G2,3 KL-(Leu)4 (D) [d)]-DMH18	$(kl)_8(kkl)_4(kll)_2kllll$	73.6 (11)	4712.51/4712.52
G2,3 KL-diPalmitamide (D) [d)]-DMH13	$(kl)_8(kkl)_4(kll)_2kkC16kC16$	128.4 (7)	4992.83/4992.84

[a)] One-letter code amino acids are used, *K* is the branching lysine residue, C-termini are carboxamide $CONH_2$, and all N-termini are free. Alkyl chains in the structure are represented by "C" followed by their number of carbon atoms. [b)] Isolated yields as trifluoroacetate salt after preparative HPLC purification. [c)] ESI-MS, see also the Supporting Information below. [d)] Peptide dendrimers previously published and characterized [1].

3. Solid phase synthesis of peptide dendrimers

Manual solid phase synthesis of peptide dendrimers. Peptide dendrimers were synthesized by placing 300 mg Rink Amide AM resin LL (0.22–0.25 mmol/g) in a 10 mL polypropylene syringe equipped as described previously. Stirring of the reaction mixture at any given step described below was performed by attaching the closed syringe to a rotating axis. The resin was swollen in DCM for 60 min. Then, the following conditions were used:

Removal of the Fmoc protecting group–At each step the Fmoc protecting group was removed with 8 mL of piperidine/DMF (1:4, *v*/*v*) for 2 × 10 min. After filtration the resin was washed with NMP (3 × 6 mL), MeOH (3 × 6 mL) and DCM (3 × 6 mL).

Coupling of the Fmoc-protected amino acids–5 eq. of Fmoc-protected amino acid, 5 eq. of Oxyma Pure (Ethyl cyano(hydroxyimino)acetate**)** and 5 eq. of DIPEA (*N*,*N*-Diisopropylethylamine) per reaction site in 8 mL of NMP/DCM (80:20, *v*/*v*) were added to the resin and the reaction was stirred for 60 min. Reactions were carried out according to the dendrimer generations with 1 h for the 0th generation, 2 h for the 1st generation, 3 h for the 2nd generation and 4 h for the 3rd generation. The resin was then washed with NMP (3 × 6 mL), MeOH (3 × 6 mL) and DCM (3 × 6 mL).

Solid phase synthesis of peptide dendrimers by CEM Liberty Blue. Peptide dendrimers were synthesized by CEM Liberty Blue (scale 0.10 mmol) using 300 mg of Rink Amide AM resin LL (0.22–0.25 mmol/g). Stirring of the reaction mixture at any given step described below was performed by bubbling of N_2 in the vial. The resin was swollen in DMF/DCM 50:50 for 15 min at R.T. Then, the following conditions were used:

Removal of the Fmoc protecting group–At each step the Fmoc protecting group was removed with 5 mL of piperidine/DMF (1:4, *v*/*v*) for 2 min at 75 °C. After filtration, the resin was washed 5 times with 5 mL DMF.

Coupling of the Fmoc-protected amino acids–5 eq. of Fmoc-protected amino acid, 5 eq. of Oxyma and 5 eq. of DIC all at a concentration of 0.2 M, were used as coupling reagents in 4 mL of DMF. The reaction was stirred for 5 minutes at 50 or 75 °C. The resin was then washed with 4 mL DMF 4 times. The couplings were repeated according to the generations and performed once for the zero generation, twice for the first generation, four times for the second generation. Only the first two peptide dendrimer generations were synthetized by CEM Liberty Blue, and the third generation synthesis was performed manually.

Deprotection of Lys(Alloc) and coupling–The resin was dried in vacuo and bubbled twice in dry DCM (8 mL) for 5 minutes with nitrogen. Solutions of $Pd(PPh_3)_4$ (0.1 eq., 10 mg) in dry DCM (3 mL) and $(CH_3)_2NH \cdot BH_3$ (25 eq., 100 mg) in dry DCM (3 mL) were added to the resin and bubbled with nitrogen for 1h. The resin was washed with dry DCM (3 × 8 mL) and reaction repeated once for 2 h. The resin was washed with sodium diethyldithiocarbamate (0.02 M in DMF, 10 mL) for 20 min and NMP, MeOH and DCM (2 × 10 mL each). Then, the carboxylic acids were coupled according to the manual procedure.

Cleavage and purification–The cleavage was carried out by treating the resins with 7 mL of a TFA/DODT/TIS/H_2O (94:2.5:2.5:1, *v*/*v*/*v*/*v*) solution for 5 h. The peptide solutions were precipitated with 40 mL of TBME, centrifuged for 10 min at 3500 rpm (twice), evaporated and dried under high vacuum for 60 min. The crude was then dissolved in a H_2O/CH_3CN mixture with 0.1% TFA, some drops of MeOH added when needed and purified by preparative RP-HPLC. The fractions of the crudes were then lyophilized. Yields are given as SPPS total yields. In all cases, yields are calculated for the corresponding TFA salts.

4. Peptide Dendrimers characterization.

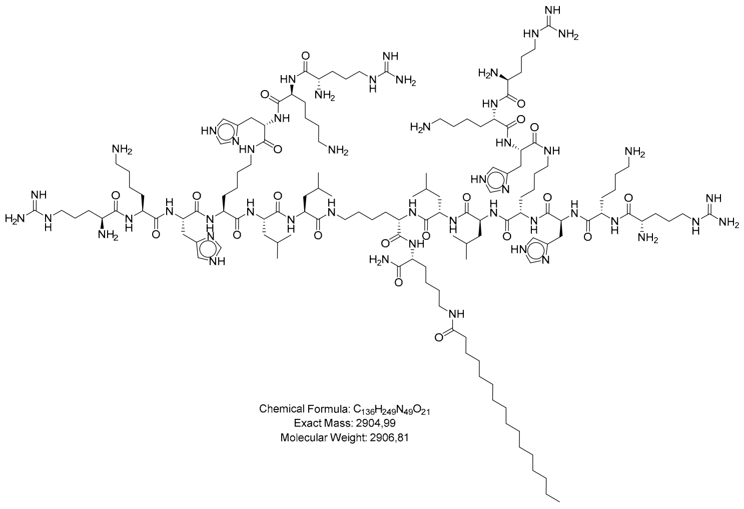

(**a**)

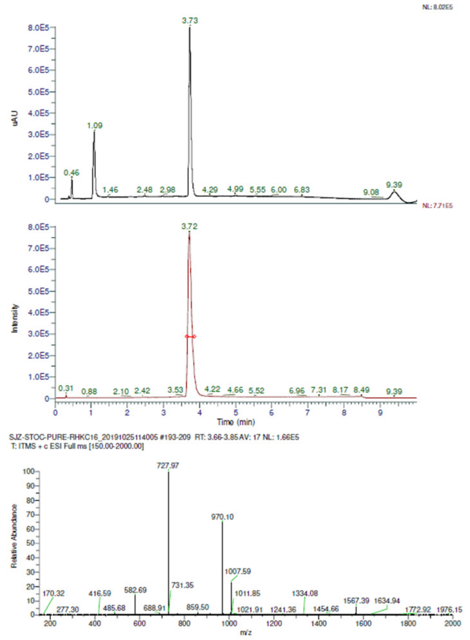

(**b**)

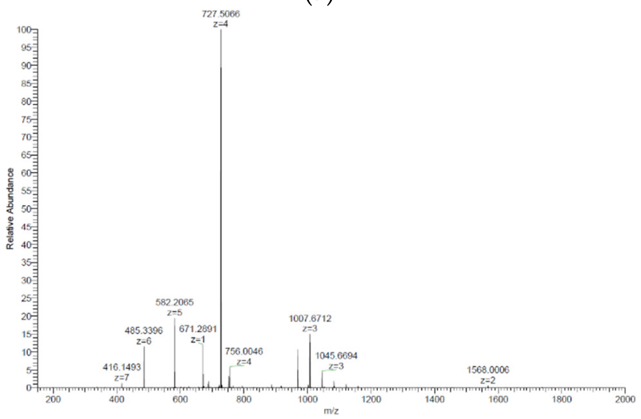

(**c**)

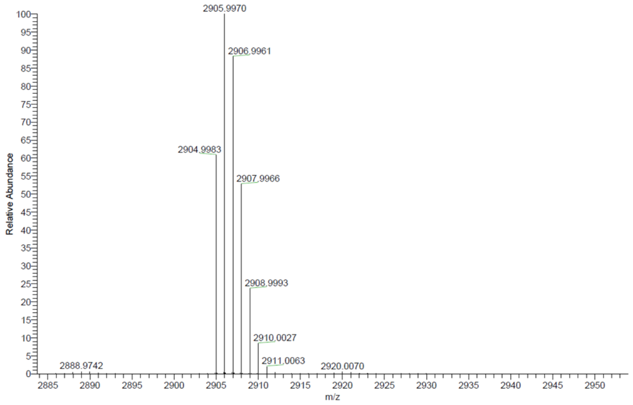

(**d**)

Figure S6. RKH-Plamitamide $(RKH)_4(KLL)_2KKC16$, it was obtained after manual synthesis as foamy colorless solid after preparative RP-HPLC (20.5 mg, 9%). (**b**) Analytical RP-HPLC: tr = 3.73 min (100% A to 100% D in 7.5 min, λ= 214 nm). (**c**), (**d**) MS (ESI+): $C_{136}H_{249}N_{49}O_{21}$ calc./obs. 2904.99/2904.99.

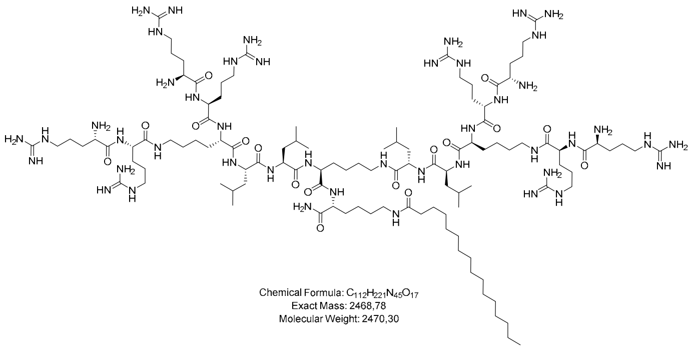

(**a**)

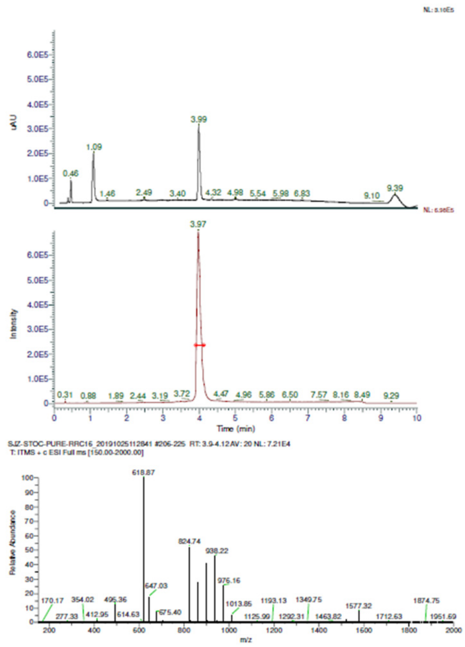

(**b**)

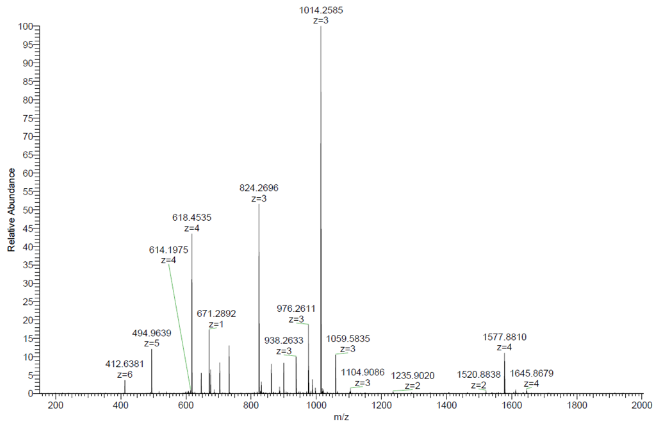

(**c**)

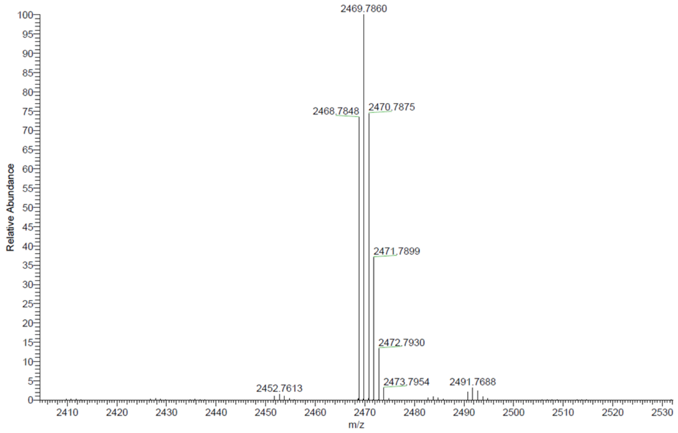

(**d**)

Figure S7. (**a**) Structure of G2 RR-Palmitamide (RR)4(KLL)2KKC16, it was obtained after manual synthesis as foamy colorless solid after preparative RP-HPLC (7 mg, 3.5%). (**b**) Analytical RP-HPLC: tr= 3.99 min (100% A to 100% D in 7.5 min, λ= 214 nm). (**c**), (**d**) MS (ESI+): $C_{112}H_{221}N_{45}O_{17}$ calc./obs. 2468.78/2468.78

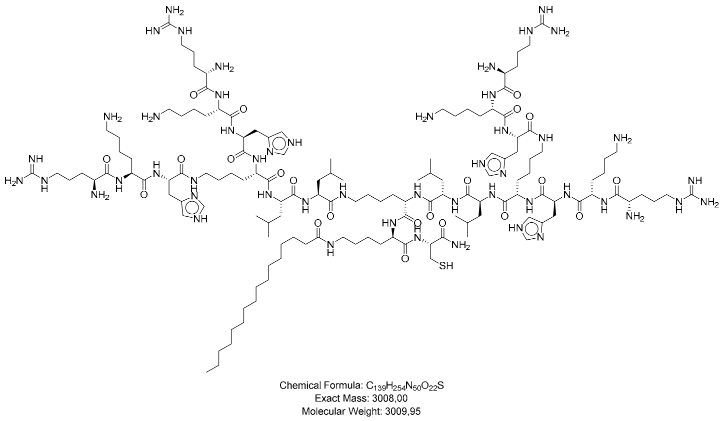

(**a**)

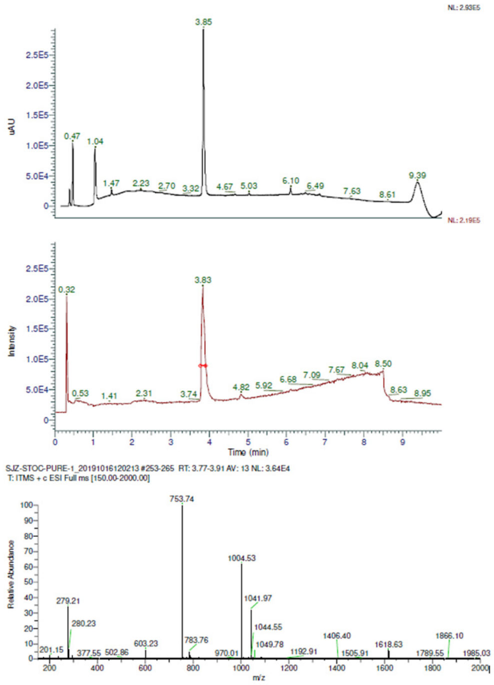

(**b**)

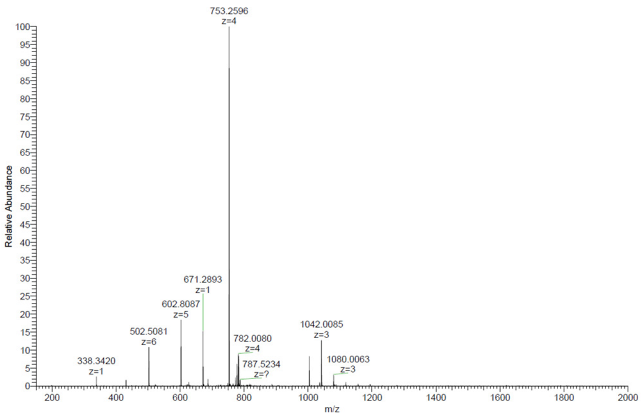

(**c**)

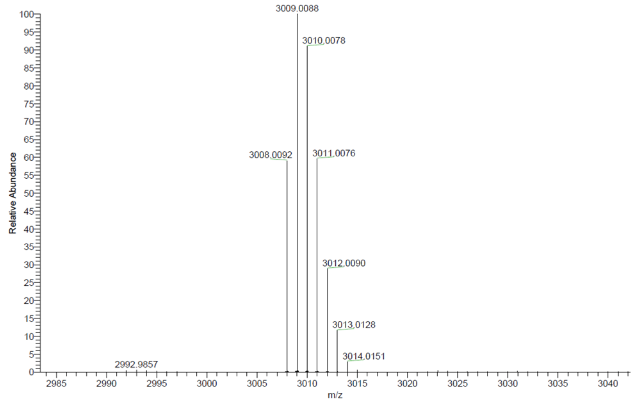

(**d**)

Figure S8. RKH-Plamitamide/Cys (RKH)4(KLL)2KKC16-C, it was obtained after manual synthesis as foamy colorless solid after preparative RP-HPLC (15.5 mg, 7%). (**b**) Analytical RP-HPLC: tr = 3.85 min (100% A to 100% D in 7.5 min, λ= 214 nm). (**c**), (**d**) MS (ESI+): $C_{139}H_{254}N_{50}O_{22}S$ calc./obs. 3008.00/3008.00.

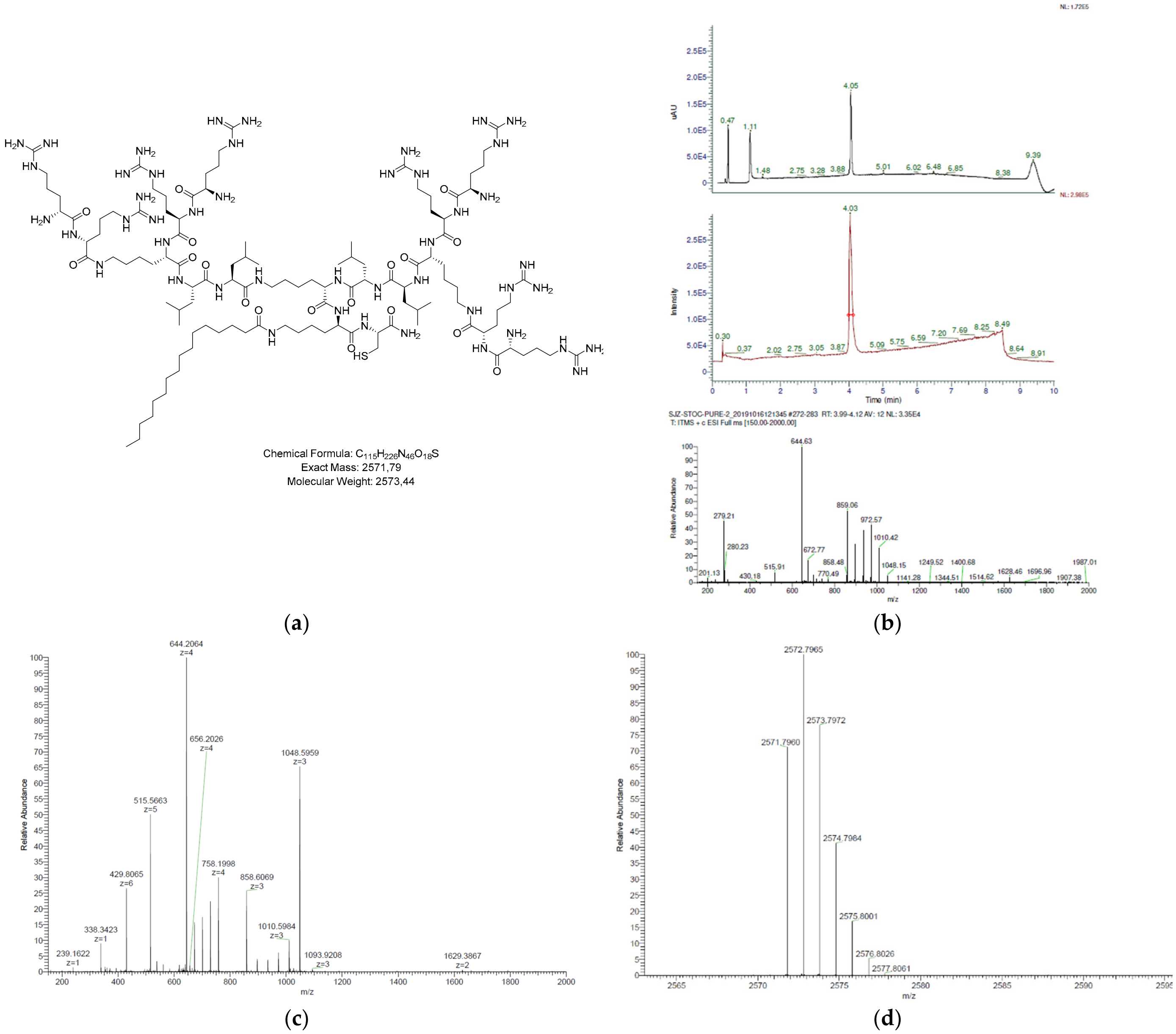

Figure S9. (**a**) Structure of G2 RR-Palmitamide/Cys $(RR)_4(KLL)_2KKC16$-C, it was obtained after manual synthesis as foamy colorless solid after preparative RP-HPLC (29.4 mg, 14%). (**b**) Analytical RP-HPLC: tr = 4.05 min (100% A to 100% D in 7.5 min, λ= 214 nm). (**c**), (**d**) MS (ESI+): $C_{115}H_{226}N_{46}O_{18}S$ calc./obs. 2571.79/ 2571.79

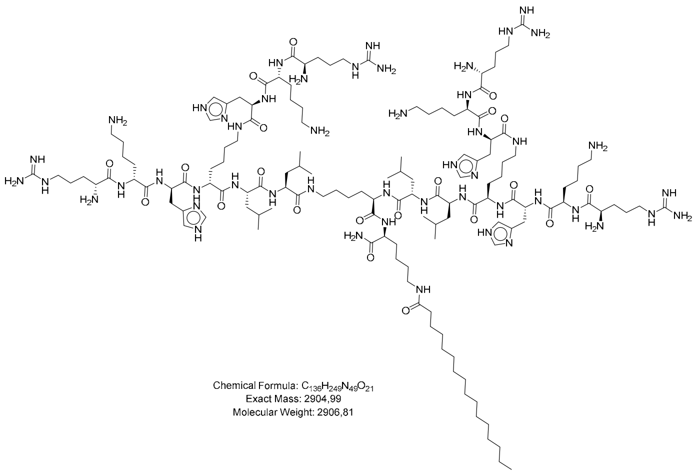

(**a**)

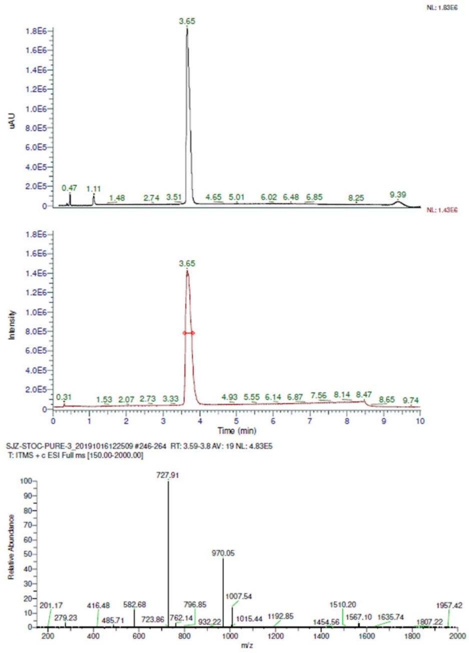

(**b**)

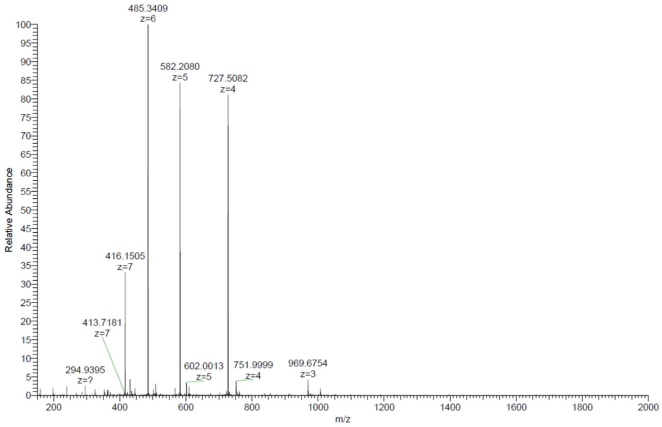

(**c**)

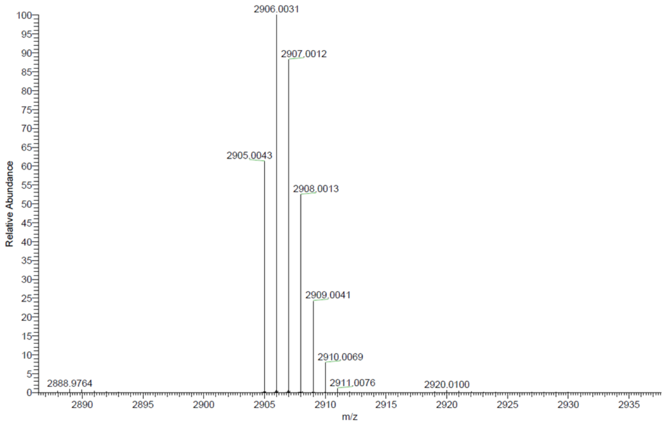

(**d**)

Figure S10. (**a**) Structure of G2 RKH-Palmitamide (D) $(rkh)_4(kll)_2kkC16$, it was obtained after manual synthesis as foamy colorless solid after preparative RP-HPLC (27.8 mg, 13%). (**b**) Analytical RP-HPLC: tr = 3.65 min (100% A to 100% D in 7.5 min, λ = 214 nm). (**c**), (**d**) MS (ESI+): $C_{136}H_{249}N_{49}O_{21}$ calc./obs. 2904.99/2904.99

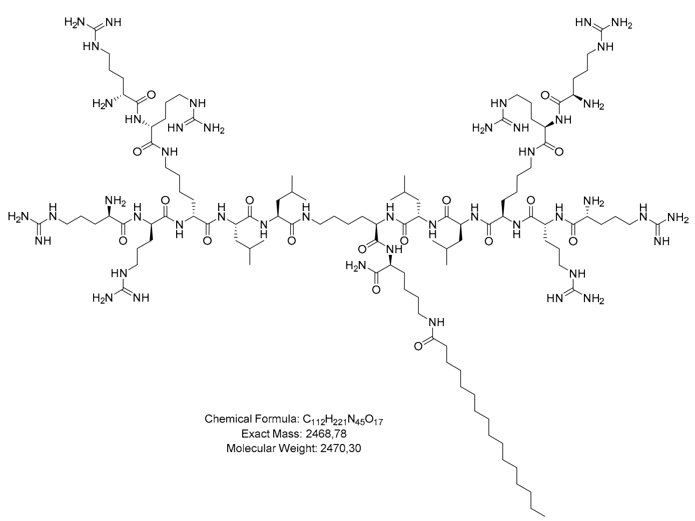

(**a**)

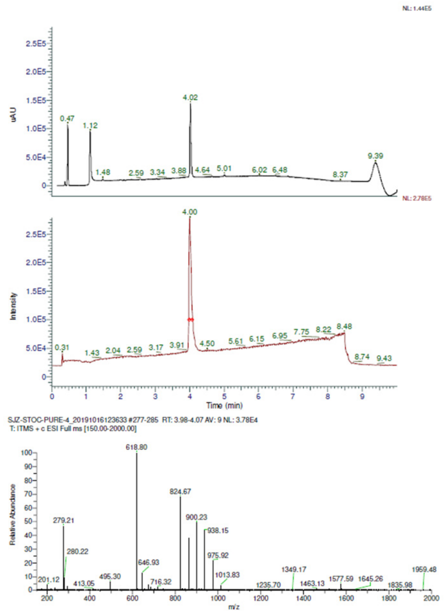

(**b**)

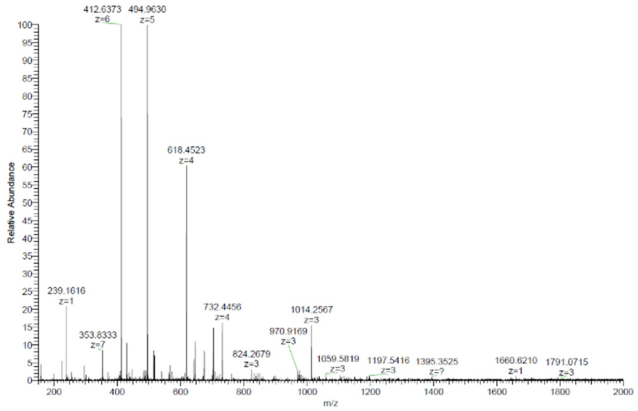

(**c**)

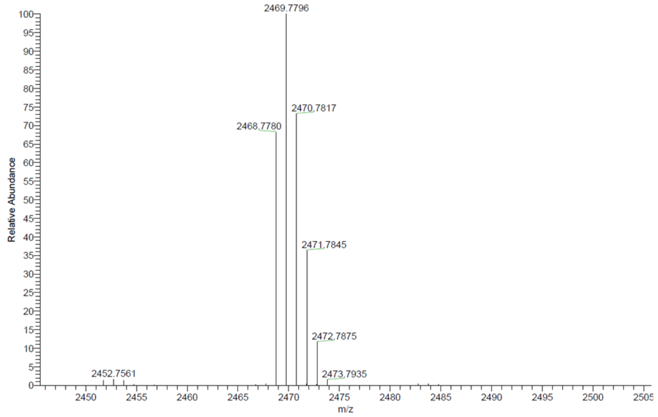

(**d**)

Figure S11. (**a**) Structure of G2 RR-Palmitamide (D) (rr)$_4$(kll)$_2$kkC16, it was obtained after manual synthesis as foamy colorless solid after preparative RP-HPLC (39.8 mg, 23%). (**b**) Analytical RP-HPLC: tr = 4.02 min (100% A to 100% D in 7.5 min, λ = 214 nm). (**c**), (**d**) MS (ESI+): $C_{112}H_{221}N_{45}O_{17}$ calc./obs. 2468.78/2468.77

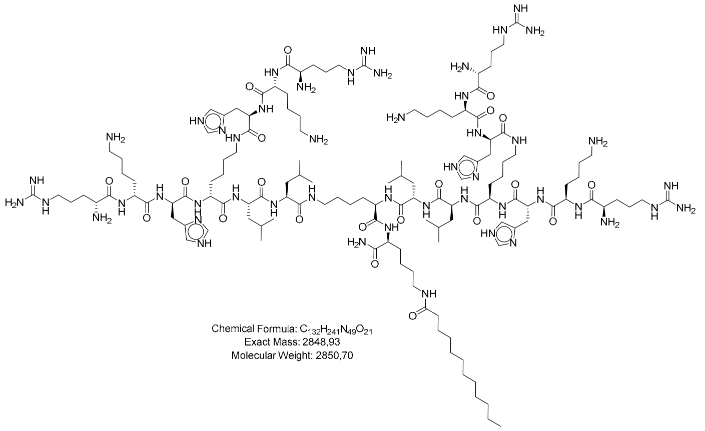

(**a**)

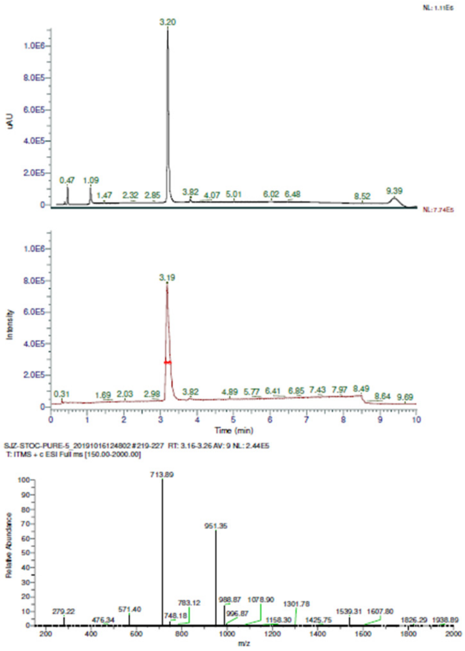

(**b**)

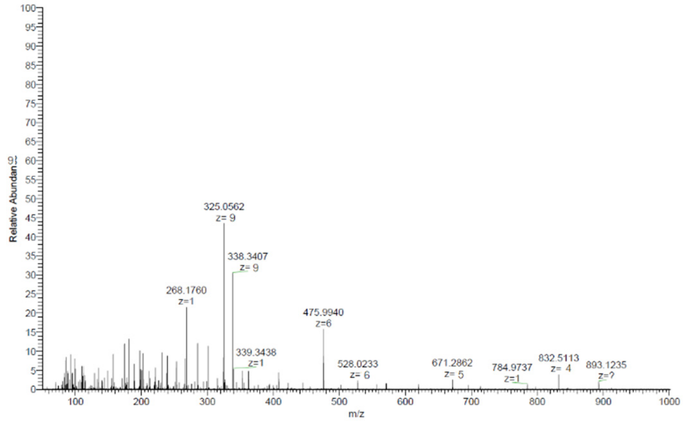

(**c**)

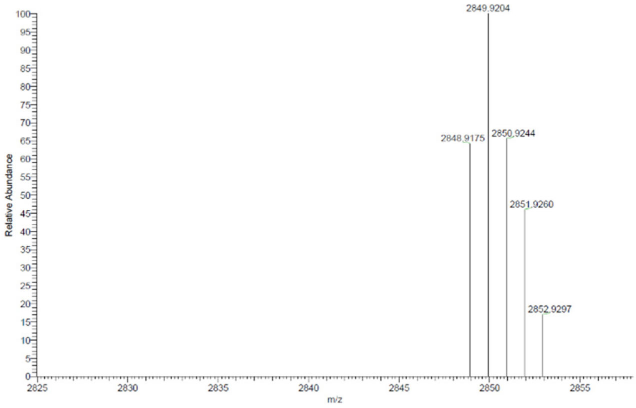

(**d**)

Figure S12. (**a**) Structure of G2 RKH-Lauramide (RKH)4(KLL)2KKC12, it was obtained after manual synthesis as foamy colorless solid after preparative RP-HPLC (12.5 mg, 5%).(**b**) Analytical RP-HPLC: tr = 3.20 min (100% A to 100% D in 7.5 min, λ = 214 nm). (**c**), (**d**) MS (ESI+): $C_{132}H_{241}N_{49}O_{21}$ calc./obs. 2848.93/2848.91

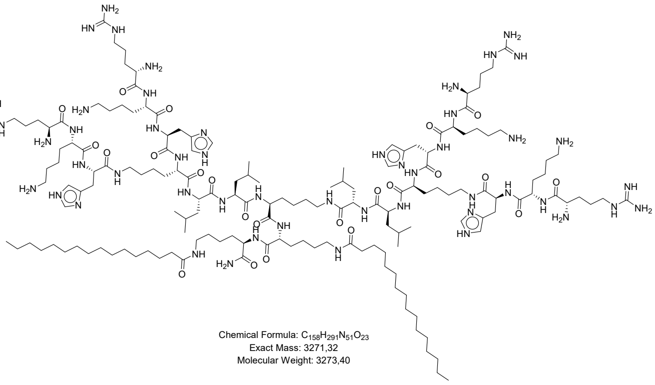

(**a**)

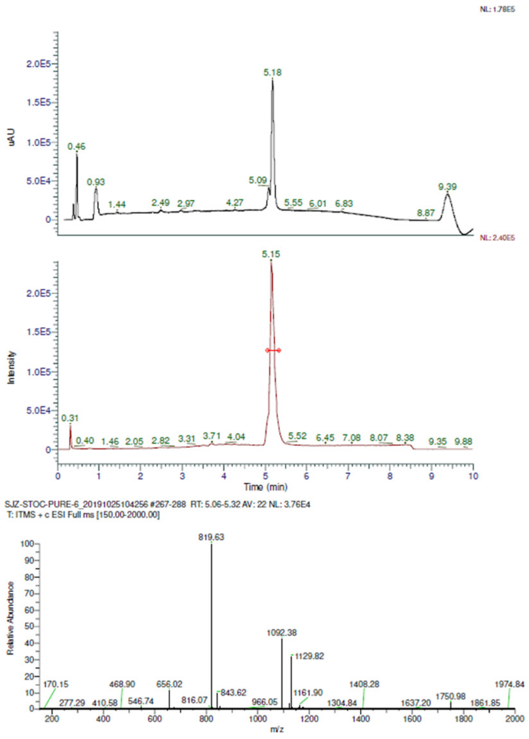

(**b**)

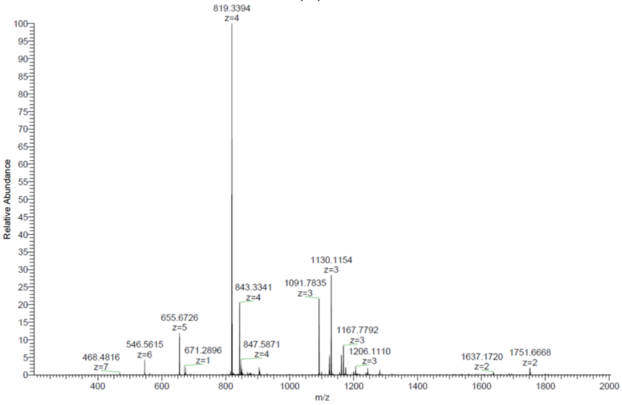

(**c**)

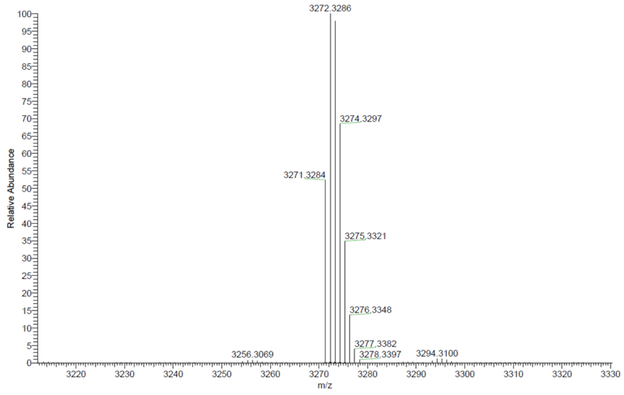

(**d**)

Figure S13. (**a**) Structure of G2 RKH-diPalmitamide $(RKH)_4(KLL)_2KKC16$-KC16, it was obtained after manual synthesis as foamy colorless solid after preparative RP-HPLC (12.2 mg, 5%). (**b**) Analytical RP-HPLC: tr = 5.18 min (100% A to 100% D in 7.5 min, λ = 214 nm). (**c**), (**d**) MS (ESI+): $C_{158}H_{291}N_{51}O_{23}$ calc./obs. 3271.32/3271.32

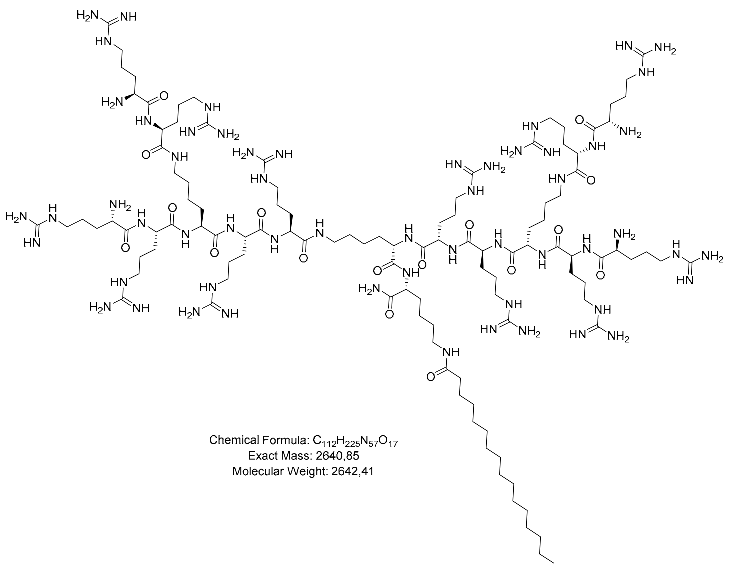

(**a**)

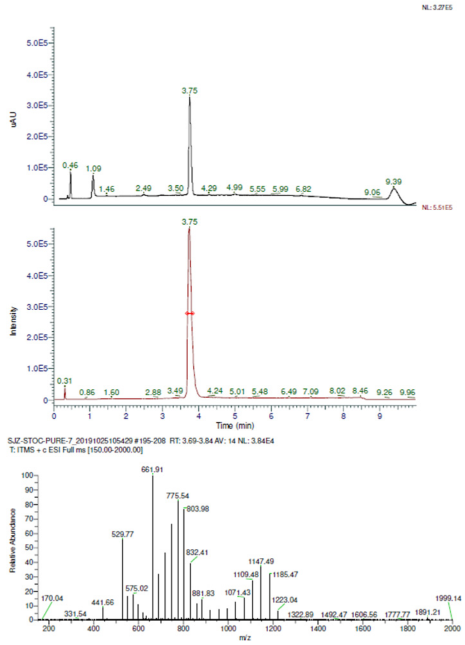

(**b**)

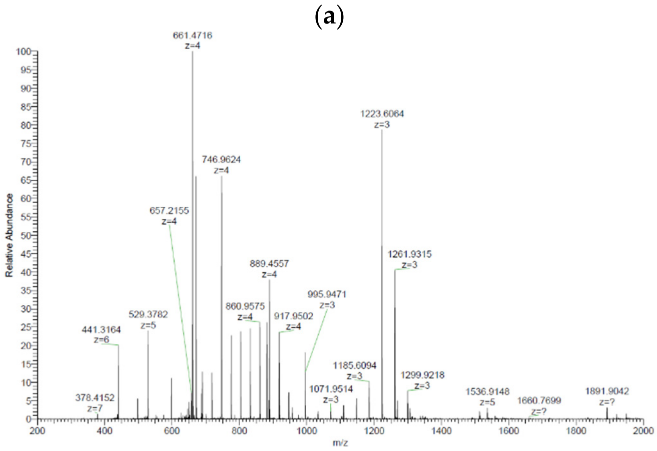

(**c**)

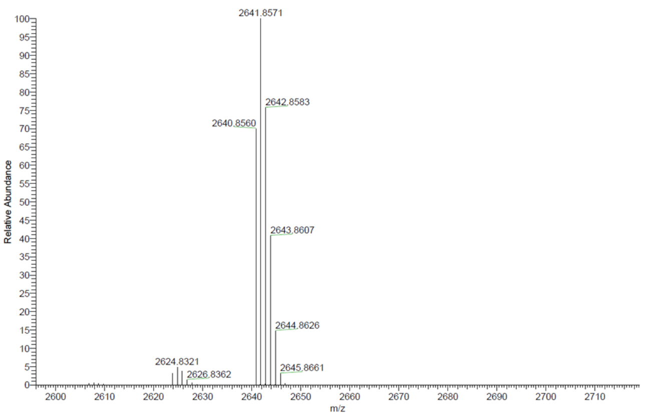

(**d**)

Figure S14. (**a**) Structure of G1,2 RR-Palimitamide $(RR)_4(KRR)_2KKC16$, it was obtained after manual synthesis as foamy colorless solid after preparative RP-HPLC (21.7 mg, 10%). (**b**) Analytical RP-HPLC: tr = 3.75 min (100% A to 100% D in 7.5 min, λ = 214 nm). (**c**), (**d**) MS (ESI+): $C_{112}H_{225}N_{57}O_{17}$ calc./obs. 2640.85/2640.85

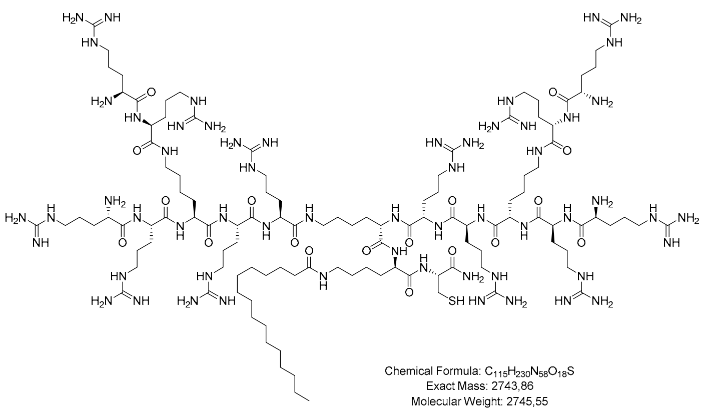

(**a**)

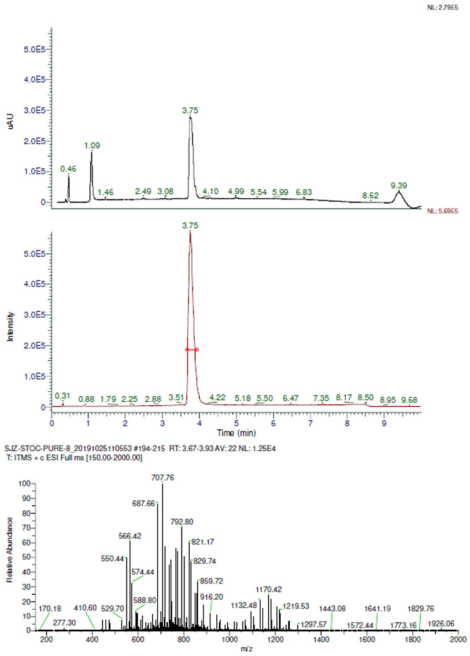

(**b**)

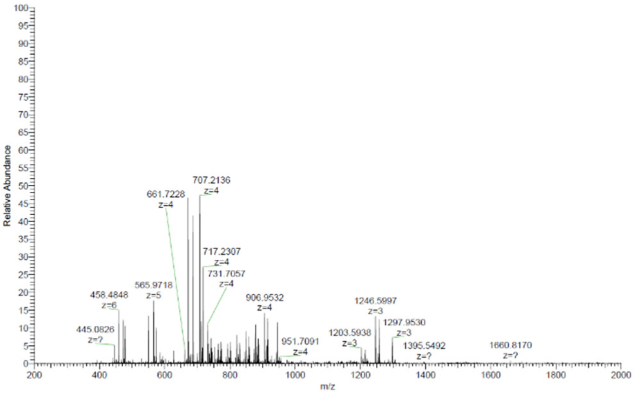

(**c**)

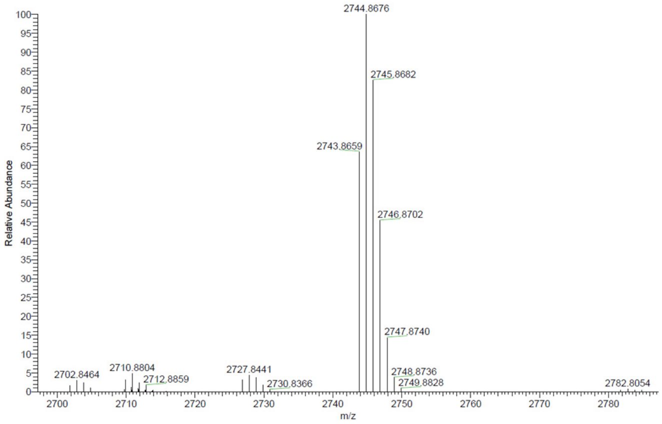

(**d**)

Figure S15. (**a**) Structure of G1,2 RR-Palmitamide/Cys (RR)$_4$(KRR)$_2$KKC16-C, it was obtained after manual synthesis as foamy colorless solid after preparative RP-HPLC (24.4 mg, 10%). (**b**) Analytical RP-HPLC: tr = 3.75 min (100% A to 100% D in 7.5 min, λ = 214 nm). (**c**), (**d**) MS (ESI+): $C_{115}H_{230}N_{58}O_{18}S$ calc./obs. 2743.86/2743.86

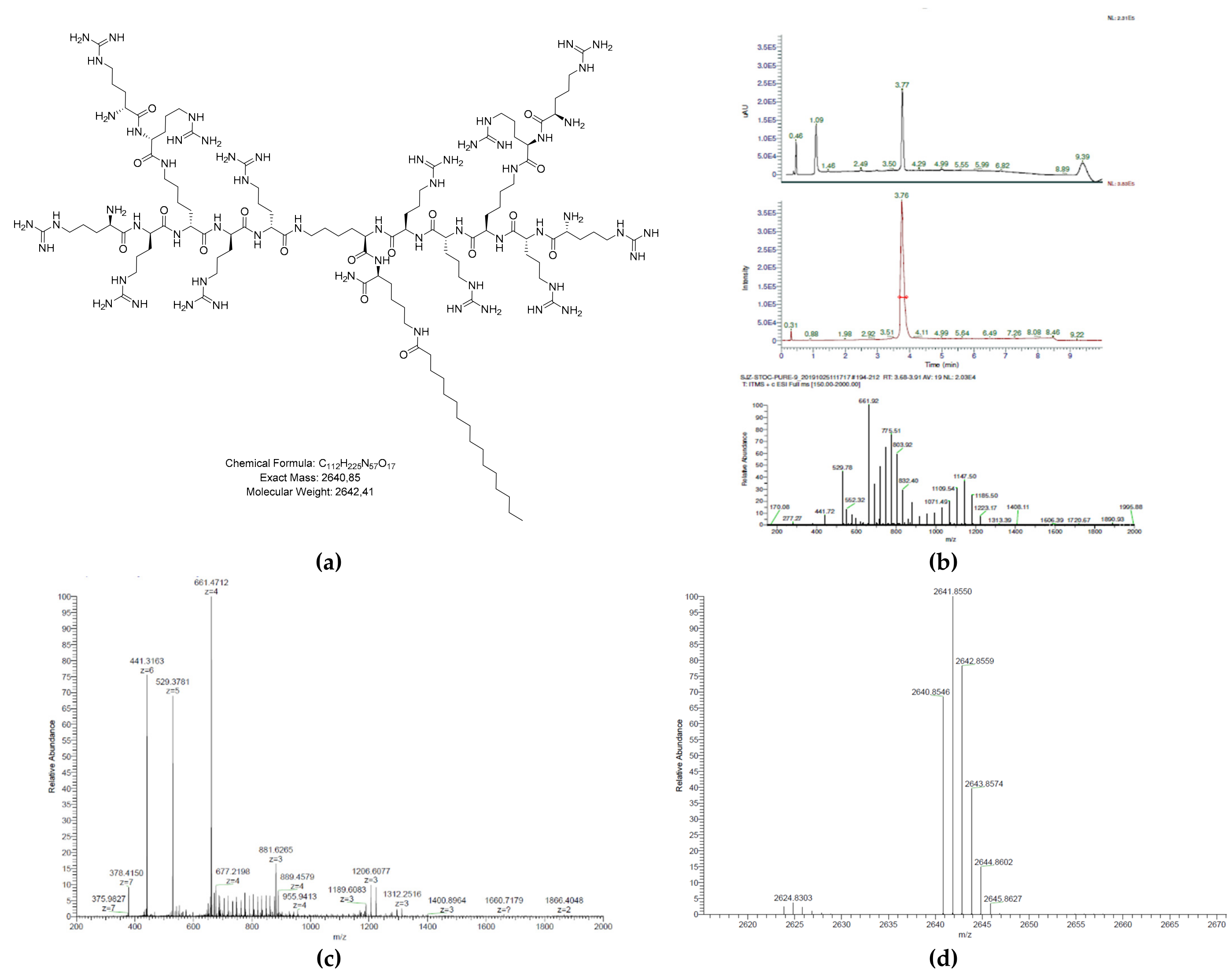

Figure S16. (**a**) Structure of G1,2 RR-Palmitamide (D) (rr)$_4$(krr)$_2$kkC16, it was obtained after manual synthesis as foamy colorless solid after preparative RP-HPLC (38.2 mg, 16%). (**b**) Analytical RP-HPLC: tr = 3.77 min (100% A to 100% D in 7.5 min, λ = 214 nm). (**c**), (**d**) MS (ESI+): $C_{112}H_{225}N_{57}O_{17}$ calc./obs. 2640.85/2640.85

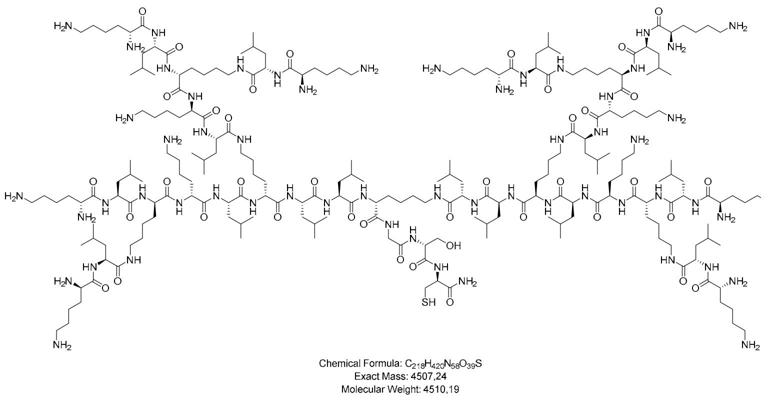

(a)

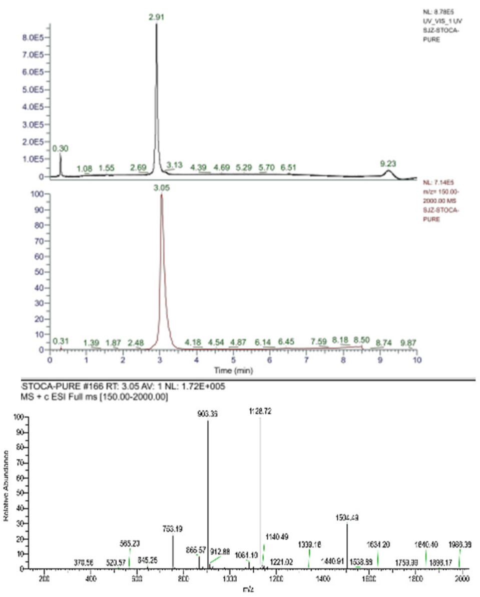

(b)

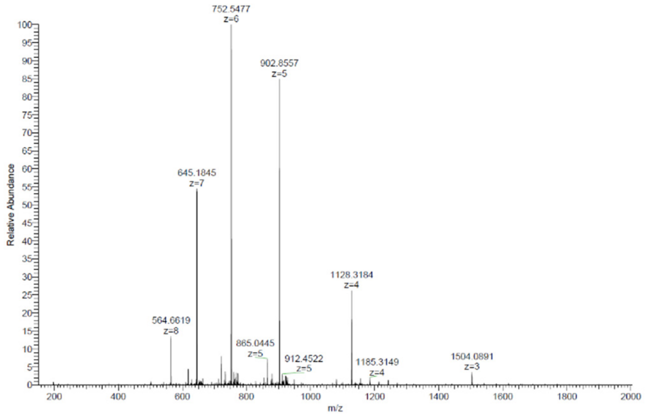

(c)

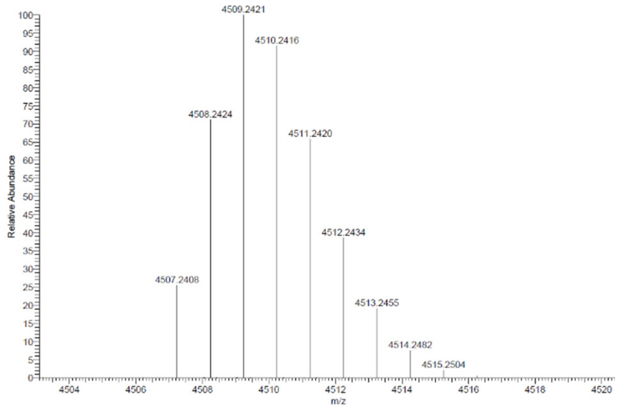

(d)

Figure S17. (**a**) Structure of G2,3 KL (D) (kl)$_8$(kkl)$_4$(kll)$_2$kgsc, it was obtained after manual synthesis as foamy colorless solid after preparative RP-HPLC (15.3 mg, 5%). (**b**) Analytical RP-HPLC: tr = 2.91 min (100% A to 100% D in 7.5 min, λ = 214 nm). (**c**), (**d**) MS (ESI+): $C_{218}H_{420}N_{58}O_{39}S$ calc./obs. 4507.24/4507.24

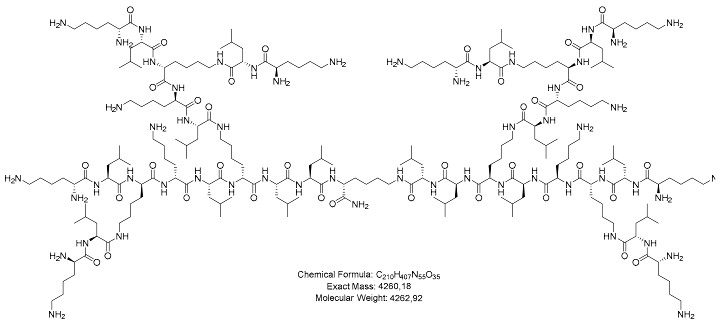

(**a**)

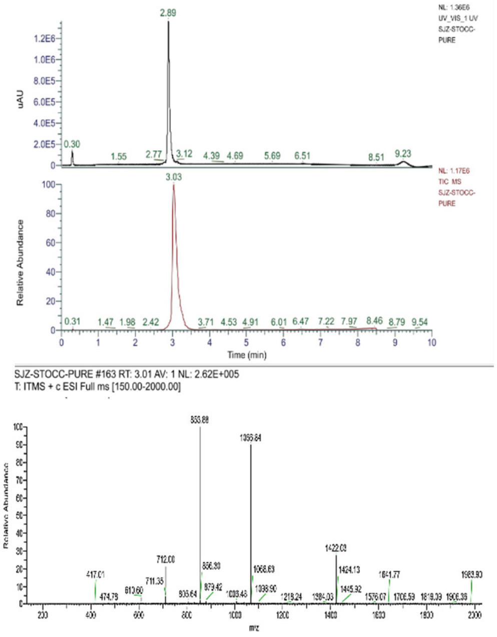

(**b**)

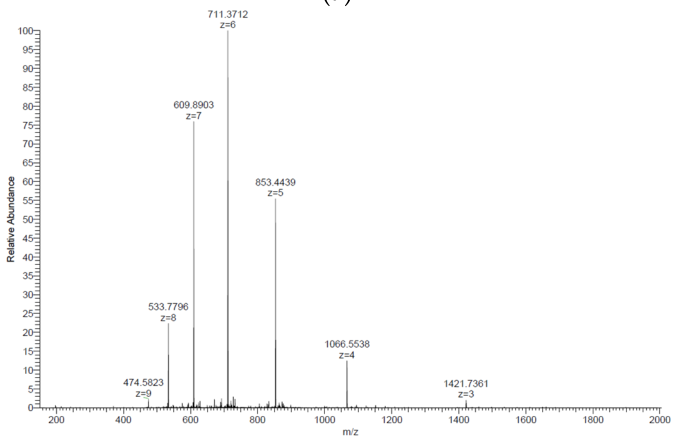

(**c**)

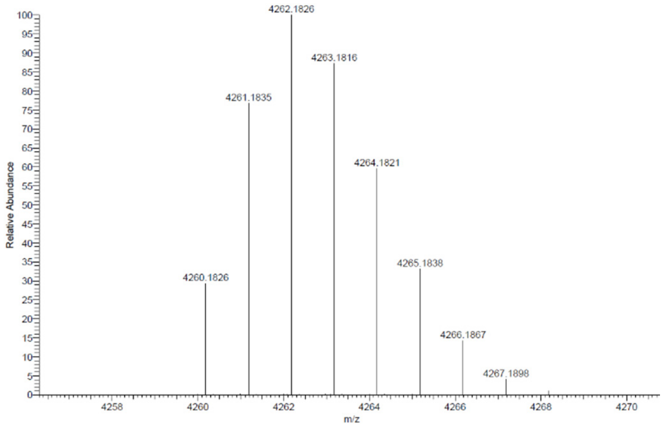

(**d**)

Figure S18. (**a**) Structure of G2,3 KL-no core (D) $(kl)_8(kkl)_4(kll)_2k$, it was obtained after manual synthesis as foamy colorless solid after preparative RP-HPLC (18.2 mg, 5%). (**b**) Analytical RP-HPLC: tr = 2.89 min (100% A to 100% D in 7.5 min, λ = 214 nm). (**c**), (**d**) MS (ESI+): $C_{210}H_{407}N_{55}O_{35}$ calc./obs. 4260.18/4260.18

References

1. Heitz, M.; Javor, S.; Darbre, T.; Reymond, J.-L. Stereoselective pH Responsive Peptide Dendrimers for siRNA Transfection. *Bioconjug. Chem.* **2019**, *30*, 2165–2182.
